# Imaging of gate-controlled suppression of superconductivity via the Meissner effect

**DOI:** 10.1038/s41467-026-75567-8

**Published:** 2026-07-18

**Authors:** K. J. Knapp, U. Ognjanović, P. J. Scheidegger, L. Ruf, S. Diesch, E. Scheer, A. Di Bernardo, C. L. Degen

**Affiliations:** 1 Department of Physics, ETH Zürich, Zürich, Switzerland; 2 Department of Physics, University of Konstanz, Konstanz, Germany; 3 Dipartimento di Fisica ‘E. R. Caianiello’, Università degli Studi di Salerno, Fisciano, Salerno Italy; 4 Quantum Center, ETH Zürich, Zürich, Switzerland

**Keywords:** Superconducting properties and materials, Imaging techniques, Superconducting devices, Scanning probe microscopy

## Abstract

It was recently discovered that supercurrents flowing through thin superconducting nanowires can be quenched by applying a gate voltage to a nearby gate electrode. This gate control of supercurrents, known as the GCS effect, could enable superconducting transistor logic. Here, we report that the GCS also manifests in a suppression of Meissner screening, establishing the phenomenon as a genuine feature of superconductivity that is not restricted to transport. Using a scanning nitrogen-vacancy (NV) magnetometer at sub-Kelvin temperatures, we image the nanoscale spatial region of GCS suppression in micron-size niobium islands. Our observations are compatible with a microscopic hot-spot model of quasiparticle generation and diffusion, and in conflict with other candidate mechanisms such as Joule heating or an electric field effect. Our work introduces an alternative means for studying quasiparticle dynamics in superconducting nanostructures, and showcases the power of local imaging techniques for understanding emergent condensed matter phenomena.

## Abstract (web summary)

Supercurrents flowing through thin superconducting nanowires can be quenched by applying a gate voltage, an effect known as gate-controlled suppression of superconductivity. Here, using a scanning nitrogen-vacancy magnetometer at sub-Kelvin temperatures, the authors report that this phenomenon also manifests in a suppression of Meissner screening and is thus not restricted to transport.

## Introduction

The recent discovery of a gate-controlled supercurrent (GCS) in superconducting nanoconstrictions^1^ has sparked significant scientific interest, owing to the unexpected observations and to potential applications in transistors for low power dissipation electronics^2–5^. A defining feature of the GCS is the complete switching of a typically small superconducting region from its zero-resistance state to the normal-resistance state under the application of a sufficiently large gate voltage. Multiple mechanisms have been proposed as the physical origin of the GCS^6^. These include a direct electric field (*E*-field) effect^1,7–9^, Joule heating^10^ and quasiparticle excitations in the superconductor. Quasiparticles, which are formed from broken Cooper pairs^11^, can be excited by electron injection^12–16^ or by high-energy phonons^17,18^ that are created through inelastic scattering of charge carriers in the substrate. The GCS could also arise from a combination of mechanisms rather than a single process^6^. Although significant work has been put into developing a microscopic understanding of the GCS effect, its precise origin is still under debate.

While the GCS effect has been studied in a multitude of transport-based experiments with various device geometries, its effect on other properties of superconductivity, such as the Meissner screening of magnetic fields, has not yet been explored. Moreover, there is a lack of spatial investigation of the GCS beyond the one-dimensional information gained from multi-gate measurements^1,16^. A recent scanning tunneling microcopy (STM) experiment varied the lateral position of the STM tip and the tip voltage to study the relation between quasiparticle injection and critical current suppression in superconducting nanowires^13^.

In this work, we report on the spatial imaging of the GCS in Nb island microstructures via the Meissner effect, complementing transport-based experiments and confirming the GCS as a genuine feature affecting superconductivity. Using scanning NV magnetometry at temperatures between 0.4 − 5.8 K, we demonstrate complete suppression of superconductivity exceeding distances of 1 *μ*m from the gate electrode. The suppression is caused by the leakage current between gate and island and depends on the dissipated power, rather than the applied gate voltage. Our results are consistent with the formation of a local hot spot at the location of highest current density, generating high-energy particles (such as phonons) that radially diffuse through the substrate and excite quasiparticles in the superconductor. These quasiparticles then locally destroy superconductivity. The measured quasiparticle diffusion length is  ~ 0.6 *μ*m and the estimated quasiparticle lifetime  ~ 10^−9^ s. We also observe a peculiar interaction between the GCS and superconducting vortices already at very low power.

## Results

### Superconducting island devices

**Fig. 1:**
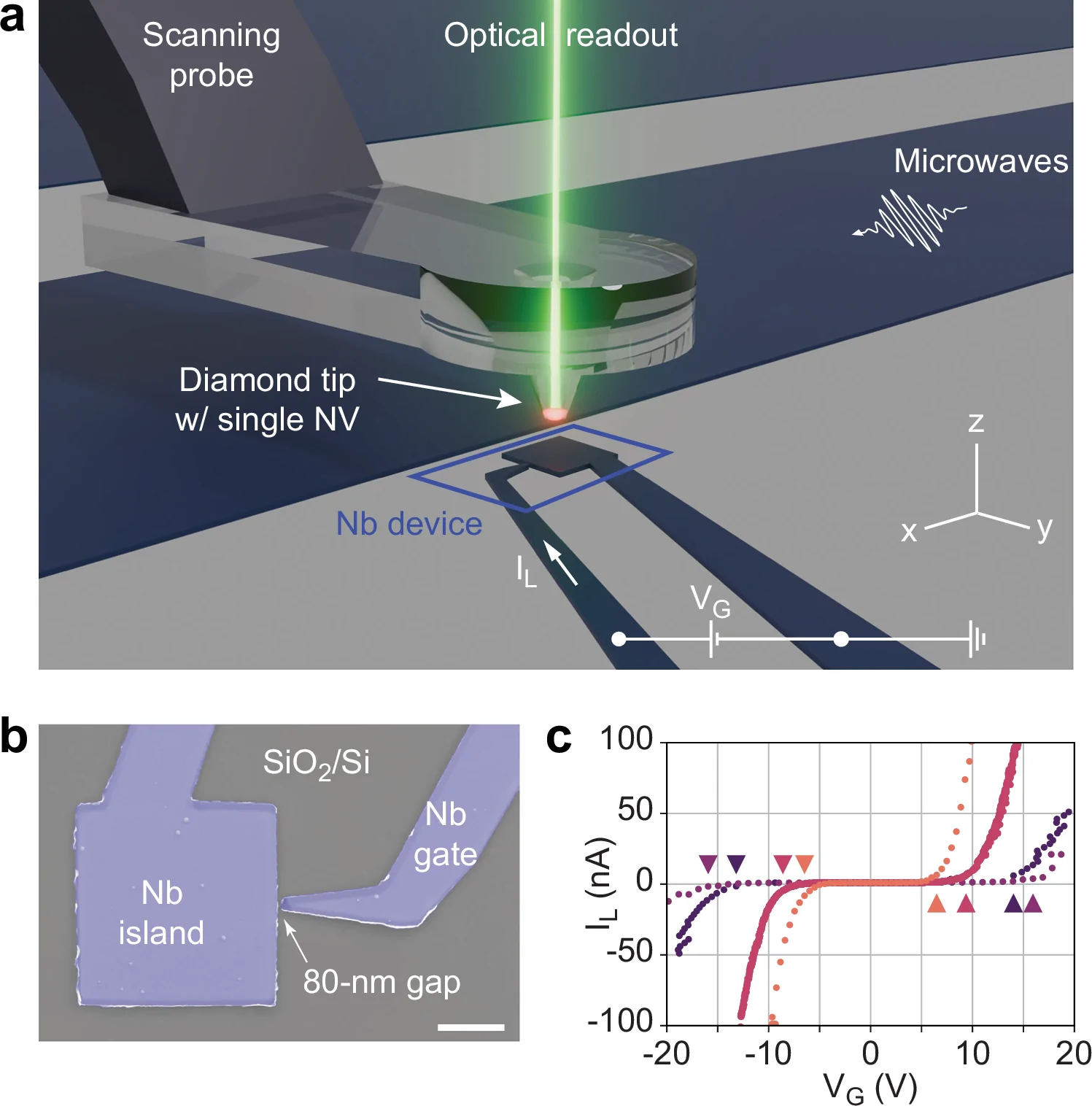
Experimental setup and superconducting devices. **a** Sketch of the scanning NV magnetometer and superconducting sample. We image the stray field above the superconducting Nb device (dark blue) by scanning with the diamond NV probe (red) at constant height (*z*_0_ ~ 100 nm). **b** False-colored scanning electron micrograph of a device identical to D2a and D2b. The superconducting Nb structure (blue) sits on top of a SiO_2_ substrate (gray). In constant voltage mode, a voltage *V*_G_ is applied between island and gate, while measuring leakage current *I*_L_. Conversely, in constant current mode, a leakage current *I*_L_ is applied while the voltage *V*_G_ is measured. Scale bar, 1 *μ*m. **c** *I*_L_/*V*_G_ curves measured on device D2b in constant voltage mode. Multiple curves (colors) are acquired over time, showing significant variation in the onset voltages of *I*_L_. The triangles mark the onset voltages ${V}_{{{{\rm{G}}}}}^{{{{\rm{ON}}}}}$ defined by the condition $P={I}_{{{{\rm{L}}}}}{V}_{{{{\rm{G}}}}}^{{{{\rm{ON}}}}}=50\,{{{\rm{nW}}}}$.

Our devices consist of approximately 20-nm-thick Nb films on top of a  ~ 5 nm Ti adhesion layer, using intrinsic Si with a 300 nm oxide layer as the substrate. The devices are patterned using a lift-off process^6^ (Methods). We fabricate three geometries in separate fabrication runs: the first geometry (device D1, Suppl. Fig. 1) is a nanowire designed to mimic transport-based GCS devices^1^. The second geometry (devices D2a and D2b, Fig. 1b) consists of a nominally 3 *μ*m × 3 *μ*m square island and a finger gate with a  ~ 80 nm gap. The third geometry (device D3, Fig. 4a) has a significantly larger Nb island with multiple gate geometries. Each of the devices is placed next to a coplanar waveguide for applying microwave pulses to the NV center magnetic field sensor^19^. The superconducting properties measured on device D1 are summarized in Supplementary Table 1.

Figure 1 c shows the leakage-current-to-gate-voltage (*I*_L_ vs. *V*_G_) characteristics for device D2b. We apply a voltage *V*_G_ between the gate and island through ohmic leads and measure the resulting leakage current *I*_L_ (Methods). We reproduce a sharp increase of *I*_L_ when reaching an onset voltage $\left\vert {V}_{{{{\rm{G}}}}}^{{{{\rm{ON}}}}}\right\vert \sim 7-16\,{{{\rm{V}}}}$ (triangles in Fig. 1c) that is typical for the onset of GCS^16^. The relatively large *I*_L_ of up to several hundred nA compared to a few nA or less for transport studies^6^ are attributed to the increased dimensions of our devices as well as to the micron-scale distances over which we probe the Meissner suppression. Interestingly, we find that ${V}_{{{{\rm{G}}}}}^{{{{\rm{ON}}}}}$ fluctuates over time, resembling recent reports of variable stress-induced leakage current in similar devices^20,21^ (Methods). In the following, we report our results in terms of the dissipated power, *P* = *I*_L_*V*_G_. We find below that the experimental hallmark of the GCS – the suppression of superconductivity – is reproducible given the same applied power *P* for all our devices.

### Qualitative observations from magnetometry scans

**Fig. 2:**
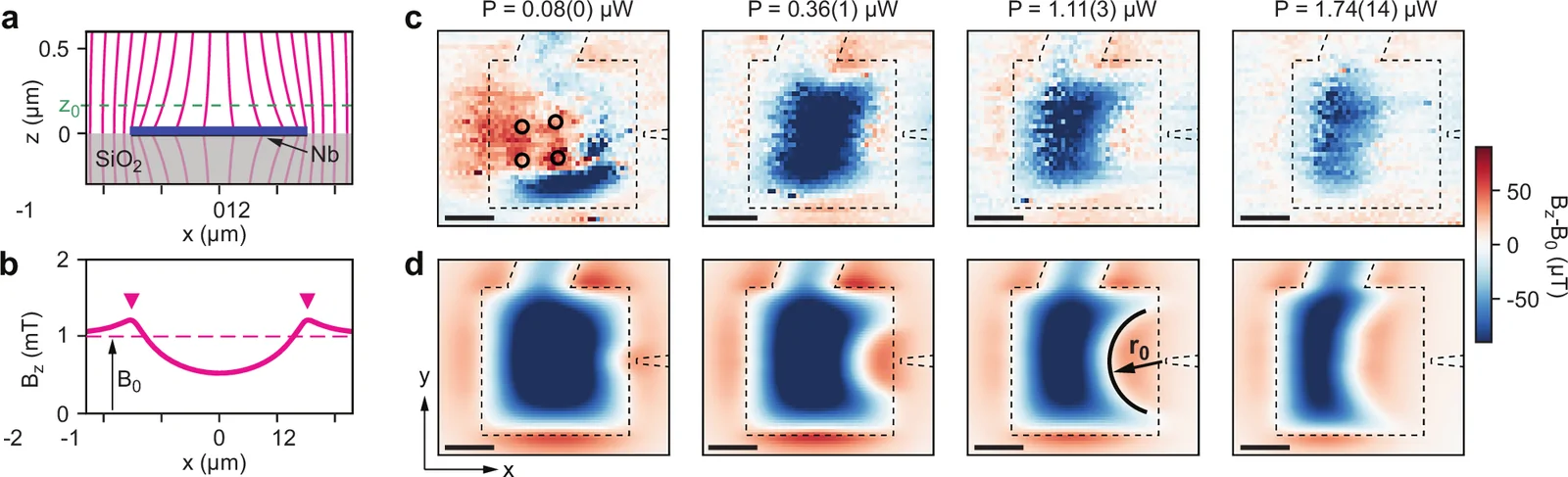
Observation of gate-controlled suppression of the Meissner effect. **a** Principle of Meissner screening in an out-of-plane magnetic field *B*_0_. The stray field lines (purple) are partially expelled from the thin Nb film (blue). The green dashed line indicates the scan height *z*_0_. **b** Model calculation of stray field at height *z*_0_. Triangles mark the field maxima that approximately coincide with the edges of the superconducting film. The dashed line is the applied background field *B*_0_. **c** Experimentally measured stray field maps plotted for increasing leakage power *P*. Blue regions of low field reflect the diamagnetic Meissner screening by the sample. Note the presence of superconducting vortices in the first panel (red *B*_*z*_ maxima) that are absent in the later panels taken at higher *P*. *B*_0_ = 1 mT. **d** Corresponding simulated magnetic field maps, assuming a circular suppression of superconductivity with radius *r*_0_ centered at the gate apex and no vortices. Simulations are performed using the SuperScreen software package (Ref. ^48^ and Methods). Scale bars in **c, d** 1 *μ*m.

To detect superconductivity in the Nb islands, we expose the devices to a small out-of-plane bias field (*B*_0_ ~ 1 mT) and image the magnetic field expulsion due to the Meissner effect. This results in lower stray field above the center of the islands and higher stray field along the edges (Fig. 2a, b)^22^. The stray field maxima roughly delineate the superconducting region. Suppression of superconductivity due to the GCS effect will then manifest as a spatial shift of the field maximum away from the gate electrode. We image the stray fields using a custom-built scanning NV magnetometer operating inside a dilution refrigerator^19^ (Fig. 1a and Methods).

We first report on qualitative measurements performed on device D2b at *T* = 0.45 K. Figure 2c shows the reconstructed *B*_*z*_ component of the measured stray field (Methods) at increasing gate power *P*. Except for the lowest power where vortices are present, discussed below, all magnetometry scans show the characteristic reduction of the stray field above the superconducting island. The gate and ohmic leads are barely resolved because their width is of order of the magnetic penetration depth (*λ* ~ 240 nm, Supplementary Table 2). For higher powers, a clear shrinking of the low-field area is observed on the right, gate-facing side of the island. Qualitatively, the imaged stray fields are in good agreement with the simulations depicted in Fig. 2d. These observations are clear evidence for a GCS effect in our Nb film and are the first key result of our study.

Curiously, near zero gate power, we observe a cluster of four superconducting vortices within the Nb island (Fig. 2c). Such vortex formation in applied bias fields is typical for thin-film superconductors^23^. Interestingly, the vortices completely vanish from the island at the onset of the GCS, even when the suppressed area is only a small fraction of the total device area (second panel in Fig. 2c). This suggests an interplay of the GCS with vortices, for example, through a modified potential at the superconducting-to-normal boundary or an interaction of quasiparticles with vortices. We briefly speculate about this feature in the outlook section.

### Quantitative analysis of quenching

Next, we establish a quantitative relationship between the gate power *P* and the spatial extent of the quenching of superconductivity. We develop two methods for quantifying the quenched region; with both methods, we assume that the quenching extends radially from the tip of the gate electrode up to a critical distance *r*_0_. This model is further justified below. In the first approach, we fit horizontal line-cuts of the measured stray field by numerical simulations of superconducting rectangles of varying width (Supplementary Note 1). In addition to giving an estimate for *r*_0_, these fits yield values of important device parameters, such as the superconducting penetration depth (Supplementary Table 2). However, because this method does not account for vortices, it cannot be applied to the entire dataset. The second approach involves the row-wise location of extrema in maps of *B*_*x*_ (D2a and D2b) or ∂*B*_*x*_/∂*x* (D3, see Suppl. Fig. 4d), which align with the edge of the superconducting domain. We quantify the quenching radius *r*_0_ by extracting the shift of these row-wise extrema relative to their position in a reference map at *V*_G_ = 0 (Supplementary Note 1).

**Fig. 3:**
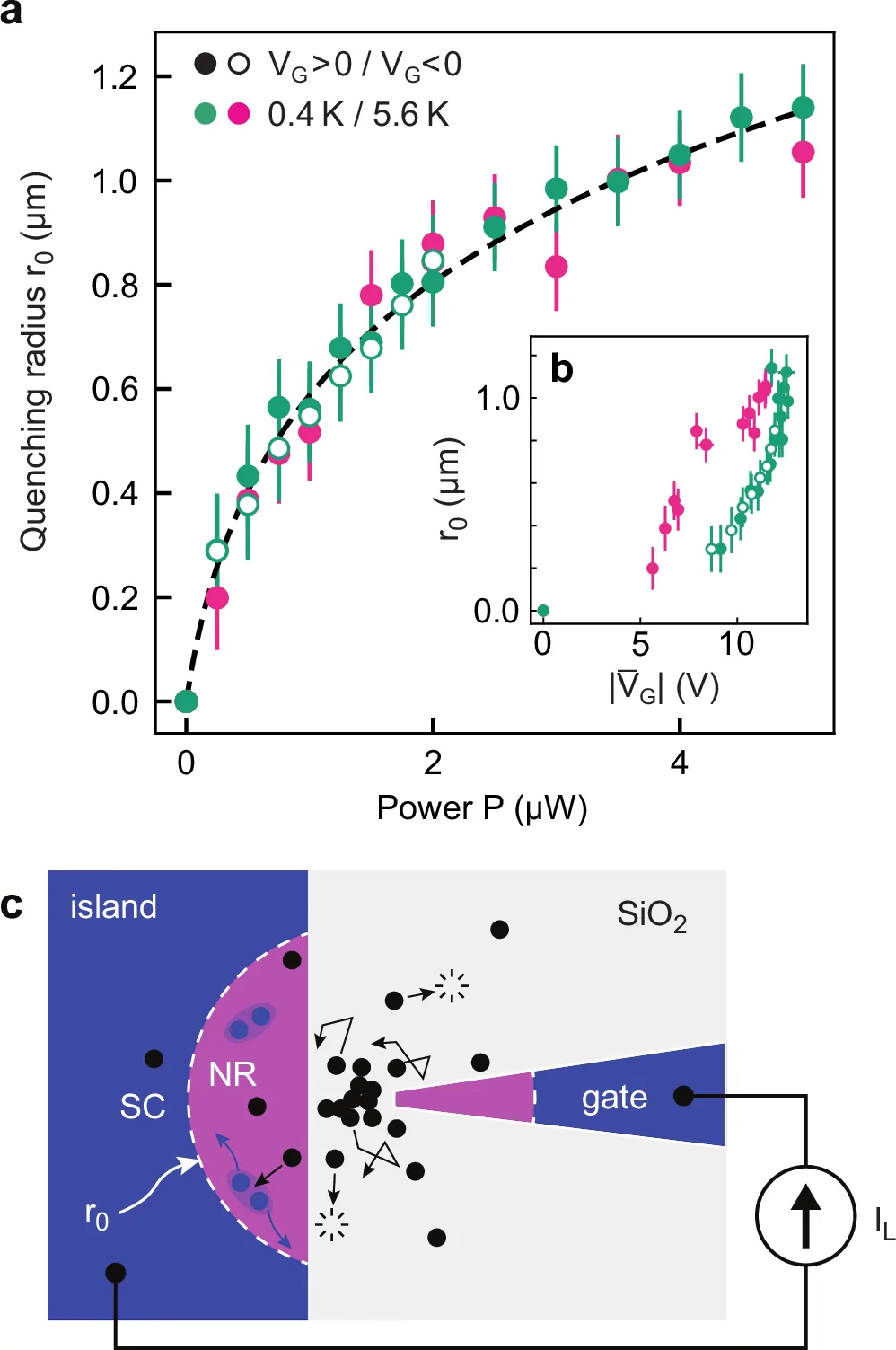
Power-dependence of GCS effect and hot-spot model of suppressed superconductivity. **a** Suppression radius *r*_0_ plotted as a function of gate power *P*, for positive and negative gate polarities (open and closed circles, respectively) and two bath temperatures (colors). Error bars indicate the standard deviation of the quenching radius *r*_0_ propagated from the experimental uncertainties using a Monte Carlo simulation (Supplementary Note 1). The dashed line is a fit to Eq. (3). Data are from device D3, gate G1. **b** Data of **a** plotted against the average gate voltage $| {\bar{V}}_{G}|$ showing only weak correlation thereby ruling out a direct field effect (see also Suppl. Fig. 4c). **c** Hot-spot model. Non-equilibrium particles (black dots), generated near the gate apex where the current density is highest, diffuse through the SiO_2_ substrate. In the Nb film, they break up Cooper pairs creating quasiparticles (blue dots), turning the superconducting (SC) into a normal resistance (NR) region within the critical radius *r*_0_.

The results of the analysis following the second approach are presented in Fig. 3a for device D3. The dataset combines measurements at two temperatures (0.4 K, 5.6 K) and at positive and negative gate voltages (i.e. both gate polarities) collected during a single cool-down. Strikingly, the quenching of superconductivity is only dependent on the gate power, and independent of temperature and gate polarity within experimental uncertainty. Moreover, the quenching of superconductivity extends over large distances exceeding 1 *μ*m at higher gate powers. Datasets from devices D2a and D2b (Figs. S3 and S4), which include a broader range of experimental parameters and measurements from several cool-downs, confirm this observation.

Figure 3 represents the second key result of our study, because the data allow us to rule out several candidate mechanisms for the gate-controlled supercurrent effect in our devices. First, Joule heating caused by the leakage current can be excluded because the quenching does not depend on temperature. Second, electron injection through vacuum is ruled out because at the high gate voltages applied in our study and given the asymmetric gate geometry, this mechanism would depend on gate polarity^6,18^. Finally, a direct electric field effect, without a leakage current, is also excluded because the same gate voltage can result in different quenching radii, shown in Suppl. Fig. 4c. This occurs when the *I*_L_ vs. *V*_G_ relationship changes. Moreover, Suppl. Fig. 8 shows two consecutive measurements that exhibit completely different suppression states at the exact same gate voltage.

### Hot-spot model

In the following, we argue that the quenching of superconductivity is caused by non-equilibrium particles (NEPs)—namely, high-energy electrons or phonons^6,13,16,17,20^, generated by the leakage current. The suggested microscopic mechanism is sketched in Fig. 3c: the NEPs, generated at the location of highest leakage current density (*i.e*., in the substrate just below the gate apex), form a local hot spot. This hot spot is similar to that observed in superconducting single photon detectors, however, it forms in the substrate and is not caused by Joule heating^24^. The number of excited NEPs can exceed the number of charge carriers of the leakage current by far^14,24^. Because of the relatively large distance between gate and Nb island, we argue that the NEPs reaching the island are mainly phonons. When impinging on the superconductor, the NEPs excite quasiparticles by breaking up Cooper pairs, leading to a suppression of superconductivity. While diffusing, both types of particles—phonons in the substrate and quasiparticles in the superconductor—also undergo relaxation, eventually limiting the radius of the suppression.

To quantify this hypothesis, we model the hot spot using a steady-state diffusion equation (Supplementary Note 2),

1 $$D{\nabla }^{2}\rho ({{{\boldsymbol{r}}}})+Q\delta ({{{\boldsymbol{r}}}})-\frac{1}{\tau }\rho ({{{\boldsymbol{r}}}})=0\,,$$

adding a point-like source term, *Qδ*(***r***), to account for excitation and a decay term, $\frac{1}{\tau }\rho ({{{\boldsymbol{r}}}})$, to account for thermal relaxation. Here, *ρ*(***r***) is the density of particles at a distance ***r*** from the source, *D* is the diffusion coefficient, *Q* is the excitation rate, and *τ* is a relaxation time. Solving for *ρ* yields the steady-state distribution of the particles,

2 $$\,\rho (r)=\frac{\alpha P\tau }{4\pi {r}_{{{{\rm{d}}}}}^{2}r}\exp \left(-\frac{r}{{r}_{{{{\rm{d}}}}}}\right)\,,$$

where *r* = ∣***r***∣ is the radial distance (assuming radial symmetry), ${r}_{{{{\rm{d}}}}}=\sqrt{D\tau }$ is the diffusion length, and *Q* = *αP*. The parameter *α* describes the creation yield of NEPs. From Eq. (2) we can deduce the power dependence of the hot-spot radius *r*_0_. For this, we assume a critical density *ρ*_c_ = *ρ*(*r*_0_) above which superconductivity is suppressed. Solving for *r*_0_, we find

3 $${r}_{0}={r}_{{{{\rm{d}}}}}{W}_{0}\left(\frac{eP}{{P}_{{{{\rm{d}}}}}}\right)\,,$$

where *W*_0_ is the Lambert-*W* function, ${P}_{{{{\rm{d}}}}}=4\pi e{r}_{{{{\rm{d}}}}}^{3}{\rho }_{{{{\rm{c}}}}}/(\alpha \tau )$ is the power for which *r*_0_ = *r*_d_ (Supplementary Note 2) and *e* = 2.718.... Eq. (3) has only two free fit parameters, *r*_d_ and *P*_d_.

Despite the simplicity of our model, which neglects the composite structure of the device, interface effects and nonequilibrium dynamics^25^, we find excellent agreement between Eq. (3) and the experimental data. Fit results are *r*_d_ = 0.57 *μ*m and *P*_d_ = 0.95 *μ*W for the dataset of device D3 (Fig. 3a), and *r*_d_ = 0.65 *μ*m and *P*_d_ = 0.36 *μ*W for the dataset of device D2b (Suppl. Fig. 4).

We can compare these values to the expected diffusion lengths in the superconductor and substrate. For quasiparticle diffusion in the superconductor, using *D* ~ 5 ⋅ 10^−4^ m^2^/s obtained from transport measurements in thin-film niobium^13^ and a quasiparticle scattering time of *τ* ~ 0.5 ns ^26^, we obtain a diffusion length $\sqrt{D\tau } \sim 0.5\,\mu {{{\rm{m}}}}$. This value is remarkably close to *r*_d_. For phonon diffusion in the substrate, we estimate *D* ~ 5 ⋅ 10^−4^ m^2^/s from the thermal conductivity and specific heat of amorphous silica at 4 K ^27,28^. Although this value is similar to that of the quasiparticles, it is likely overestimating the phonon contribution to diffusion. First, phonon scattering is strongly enhanced for the high-energy phonons^29^ that are required to break up Cooper pairs. Second, the crystalline Si substrate underneath the oxide layer has a long phonon mean-free path^17^ and serves as an efficient heat sink. Both processes work to reduce lateral phonon diffusion. Therefore we conclude that while both phonons and quasiparticles contribute to the overall diffusion, the latter are dominating the diffusion process.

Looking forward, follow-up experiments will further investigate the role of the substrate^30^. If there were a strong phonon contribution to diffusion of the NEPs, the use of a material with a high thermal diffusivity *D*, such as diamond or AlN, should increase ${r}_{{{{\rm{d}}}}}\propto \sqrt{D}$ and ${P}_{{{{\rm{d}}}}}\propto \sqrt{{D}^{3}}$. Likewise, the injection efficiency would be modified. A proper theoretical model will also account for interface effects and the exact phonon density of states by solving the Boltzmann transport equation (BTE)^25,31,32^. Finally, a future experiment could aim at measuring the quasiparticle relaxation time directly using time-resolved magnetometry^33,34^.

### Dependence on gate geometry

**Fig. 4:**
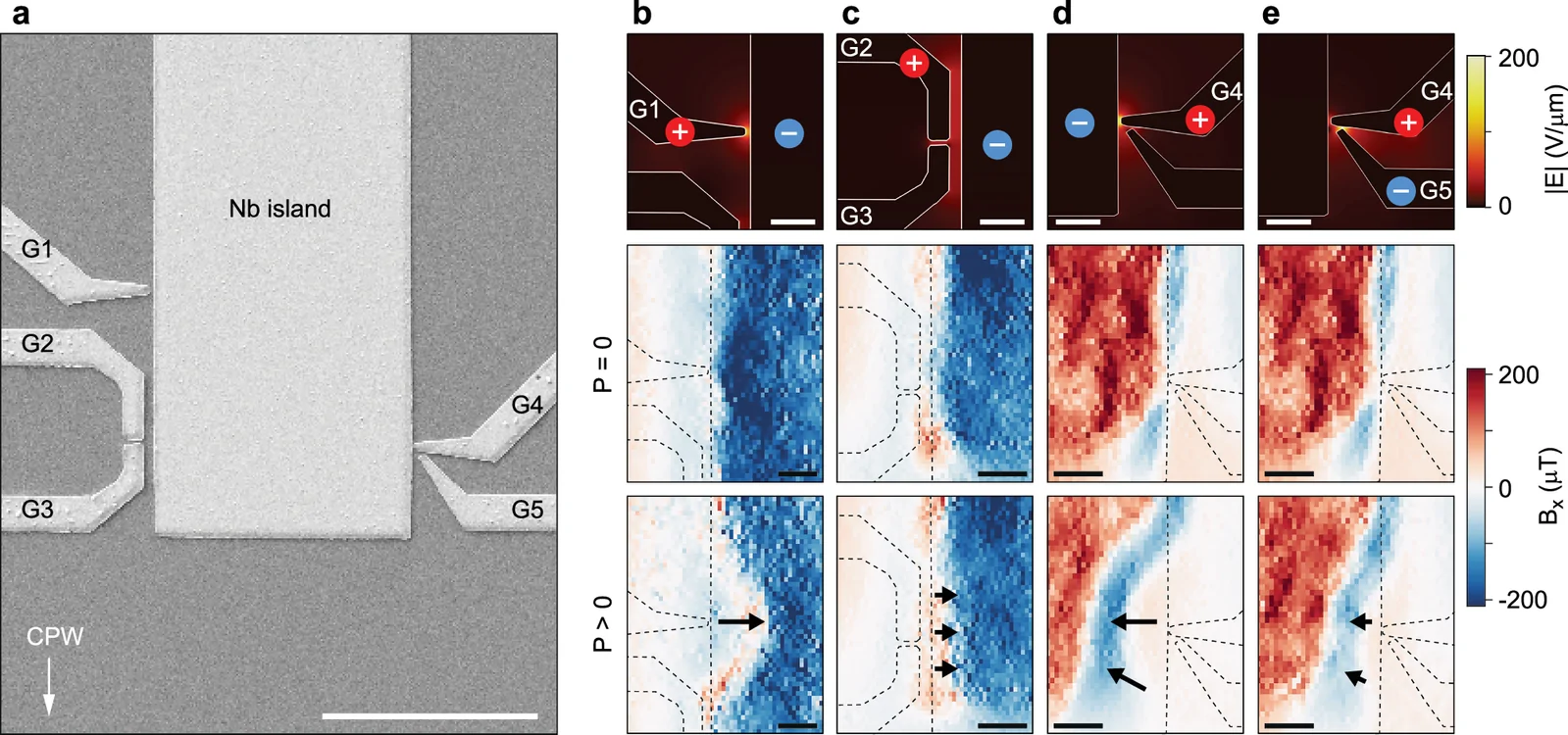
Suppression of superconductivity for various gate geometries. **a** Scanning electron micrograph of device D3. Scale bar, 5 *μ*m. **b**–**e** Electrode configurations. The top row shows a finite element modelling (FEM) simulation of the electric field strength at *V*_G_ = 10 V. The middle row shows the magnetometry map (*B*_*x*_) for zero gate voltage (*P* = 0). The bottom row shows the corresponding magnetometry map for non-zero gate power (5 *μ*W in **b**, 2 *μ*W in **c**–**e**). Dotted contours indicate approximate boundaries of the Nb island and gates (horizontally shifted to align with the stray field edge for clarity). Arrows indicate suppression with applied power. Note that the stray field is incompletely screened by the superconductor, presumably due to the presence of vortices and non-superconducting grains. This leads to regions of negative *B*_*x*_ (blue in **b**, **c**) or positive *B*_*x*_ (red in **d**, **e**) above the Nb island, respectively. See Suppl. Fig. 5 for an overview scan of the entire island. Scale bars, 1 *μ*m.

Thus far, we have investigated the traditional side-gate geometry where the finger gate is within 100 nm from the large superconducting island. In Fig. 4, we extend the study to different gate geometries including gates at larger distances and extended gate electrodes (Fig. 4a). Transport experiments with similar geometries have indicated a GCS effect even for these situations^17^.

Figure 4b shows quenching for the original finger-gate geometry (G1), placed far from the upper and lower corners of the device to eliminate boundary effects. The electric field—and hence the current density—is concentrated in the region between the gate apex and island, effectively forming a point-like source on the scale of the observed quenching. The suppression takes a circular shape at all imaged gate powers *P* (Suppl. Fig. 6). This justifies our earlier assumption of a point-like injection of NEPs and a radially symmetric hot spot.

Next, we investigate the extended gate electrode (G2). Here, quenching is barely noticeable (Fig. 4c) and is elongated along the length of gates (G2) and (G3). This makes intuitive sense: because the electric field extends along the  ~ 2.6 *μ*m length of the electrodes (G2) and (G3), the current density is lower for the same gate power. This results in an elongated quenching region and a shorter quenching distance.

Figure 4d shows GCS quenching for a finger gate (G4) close to the lower right corner of the device. Here, a distinct corner effect becomes apparent. (A similar effect is visible for higher powers in Fig. 2c). The corner effect can be explained by the relatively long penetration depth that smoothens sharp features. Finally, in Fig. 4e, we apply a voltage between (G4)-(G5) with the Nb island floating. Strikingly, and in agreement with Ref. ^17^, quenching occurs even in this situation. However, the quenched area is reduced with respect to Fig. 4d, consistent with a hot spot that is removed by  ~ 0.8 *μ*m from the island’s edge compared to Fig. 4d. Again, the observation is compatible with a point-like injection and subsequent diffusion of NEPs, where the effective distance to the injection site governs the extent of quenching.

## Discussion

Our experiments show a reproducible suppression of the Meissner screening in Nb microstructures under the application of power to an adjacent gate. The range of full suppression is larger than 1 *μ*m and exceeds previous studies of nanowires by at least a factor of two^6^. Required gate powers are  ~  *μ*W and several orders of magnitude higher compared to transport measurements^6^. This result is intriguing, and while it can be partially explained by the extended size of our Nb microstructure, it may hint at differences in the GCS in transport versus the GCS related to Meissner screening.

Future studies will investigate the interaction between superconducting vortices and the GCS. Interestingly, as visible in Fig. 2c, the vortices completely escape the microstructure already at low gate voltages that are more typical for transport GCS. Moreover, while all measurements show interactions between vortices and the GCS, the escape power is higher in the dataset acquired at 3.5 K (Suppl. Fig. 7) compared to the ones at 0.45 K (Fig. 2c) and 5.8 K (Suppl. Fig. 7). The reasons for such GCS-vortex interactions are unknown and motivate further study. Previous work on superconductors showed the movement of vortices due to applied current^23^ or magnetic force^35,36^, and the attraction of vortices to thermal hot spots^37^ or regions with an enlarged quasiparticle population^38^.

Finally, our experiments highlight some genuine advantages of scanning magnetic imaging techniques. They enable investigation of the GCS effect with nanoscale resolution and over a temperature range from well below the superconducting gap to above the critical temperature of Nb. Future studies may further correlate the magnetic images with electric field maps^39^, noise measurements^40^, thermal mapping^41,42^ or time-resolved sensing^34^.

## Methods

### Device fabrication

Nb devices (thickness  ~ 20 nm) with a Ti ( ~ 5 nm) adhesion layer were patterned on a polished and chemically-cleaned 350 *μ*m-thick (100) Si substrate with a 300 nm wet/dry/wet SiO_2_ top layer (MicroChemicals). A  ~ 225 nm layer of poly(methyl methacrylate) positive resist (PMMA, Kayaku 950 A4) was spin-coated onto the substrate and baked on a hot plate at 180 ^∘^C for 90 s. Full waveguide and device patterning was performed in a single step using electron beam lithography (20 kV,  ~ 300 *μ*C/cm^2^ dose). The positive resist was developed in a solution of methyl isobutyl ketone (MIBK) and isopropanol (IPA). Nb and Ti films were deposited using RF magnetron sputtering (Ar flow: 17 sccm, pressure: 1.5 mTorr). Ti was deposited using a single RF gun at 200 W with a deposition rate of  ~ 0.05 nm/s. Nb was deposited using two RF guns and one DC gun, achieving a rate of  ~ 0.4 nm/s. After deposition, the devices were immersed in a 50 ^∘^C acetone bath for several hours, followed by brief ultrasonication (a few seconds) for lifting off the mask. They were then rinsed with IPA and dried in a N_2_ stream.

### Electrical measurements

The devices’ electric properties were characterized using a Keithley 2450 source measure unit (SMU). The SMU was used in either a constant voltage or constant current mode setting. The *I*_L_ vs. *V*_G_ characteristics shown in Fig. 1c were acquired in constant voltage mode, in order to enforce a uniform sampling of the voltage range. By contrast, all magnetometry scans used the constant current mode setting. In addition, we implemented a constant power mode on top of the constant current mode using a software proportional-integral-derivative (PID) control loop. In this constant power mode, the gate voltage was continuously measured and the SMU current setpoint adjusted such that the power *P* = *I*_L_*V*_G_ remained constant. This software constant power mode was used in the measurements on device D3.

### Leakage currents

Since we measured the current and voltage at the source, the measured current include both the sample leakage and contributions from the wiring between the voltage source and the device. At low bias voltages (∣*V*_G_∣ ≲ 4 V), the current scaled linearly with voltage, corresponding to an ohmic resistance of  ~ 60 GΩ, which we attributed to parasitic leakage in the wiring or connectors^16^. At higher gate voltages, the current increased exponentially and eventually dominated over the wiring contribution.

The leakage currents measured in our experiments showed a behavior similar to that reported from variable stress-induced leakage currents (vSILCs)^6,20^. Firstly, the onset voltages observed in the *I*_L_/*V*_G_ characteristics varied from measurement to measurement, as shown in Fig. 1c. Moreover, we observed that the gate voltage (or gate power) varied over the course of hours while operating the SMU in constant current mode (Suppl. Fig. 2). Other possible origins of *V*_G_ variations include charge accumulation and capture in the gate dielectric giving an additional capacitance, involvement of surface traps that are sensitive to the enviroment (*e.g*., air exposure), and the onset of filament formation due to high electrical stress^43^.

### Magnetometry measurements

The magnetometry images were acquired using modified atomic force/confocal microscope from Attocube, integrated into the cold insertable probe of a Leiden Cryogenics CF-CS100 dry dilution refrigerator. Details of the setup can be found in Refs. ^19,44^. For scanning, we used a commercial NV scanning probe from QZabre. The tuning fork was operated in non-contact atomic force microscopy (AFM) mode with a NV-to-sample separation of approximately *z*_0_ ~ 100 nm. Measurements were performed using several NV probes, all cut along the 〈100〉 crystal orientation.

Static out-of-plane magnetic fields were applied using a superconducting vector magnet (American Magnetics Inc.). The projection of the magnetic field along the NV quantization axis *B*_NV_ was measured by determining the frequency of the *m*_s_ = 0 to *m*_s_ = − 1 spin transition, *f*_0,−1_, using the pulsed ODMR technique^45^. *B*_NV_ was obtained by comparing the resonance frequency *f*_0,−1_ to the zero-field resonance *f*_ZFS_ via the relation *B*_NV_ = 2*π*(*f*_ZFS_ − *f*_0,−1_)/*γ*_e_, where *γ*_e_/2*π* = 28.025 MHz/mT is the gyromagnetic ratio of the electron. The projection axis of *B*_NV_ is given by the crystallographic orientation of the single crystal diamond tip, and is approximately *θ* = 54^∘^ and *ϕ* = {0^∘^, 90^∘^, 180^∘^, 270^∘^}, where *θ* and *ϕ* are the polar and azimuth angle in the scanning frame of reference. Typical magnetic field sensitivities at cryogenic temperatures were between $\sim 10-20\,\mu {{{\rm{T}}}}/\sqrt{{{{\rm{Hz}}}}}$. Finally, we applied a Fourier transform-based algorithm^46^ to reconstruct the three-component vector field ${{{\bf{B}}}}=\left({B}_{x},{B}_{y},{B}_{z}\right)$ from the measured *B*_NV_ projection.

To read out the spin state of the NV optically, an in-house-built green laser (520 nm^44^) is shone on the NV and the fluorescence is collected. Consequently, due to the size of the focused beam and the proximity of the NV to the sample, some laser light reaches the sample. This can be observed from the heating of the sample when operating at  mK temperatures. However, the heating power of the microwave pulses ( ~ 300 *μ*W) is significantly larger than the laser power when pulsed ( ~ 50 *μ*W)^19^). Generally, the laser is only on for a short duration of time ( ~ 1 μs) as the optically-detected magnetic resonance (ODMR) sequence is performed in a pulsed manner. The laser initialization/readout pulse and the microwave *π*-pulse are separated by a dark interval of  ~ 1 μs in our pulse sequence, while quasiparticle relaxation times are on the order of  < 1 ns ^47^. This means that the superconductor has sufficient time to recover from the previous illumination and is effectively not affected besides the global heating, which is well understood^19^. Additionally, because Meissner screening of the full device has been imaged and we only observe quenching once a sufficient power *P* is applied (see Fig. 2 and Fig. 3), the invasiveness of the measurement in our case is insufficient to break superconductivity.

### Magnetic field simulations

Simulations of the magnetic stray field were carried out using the SuperScreen software package^48^. For this, we assumed a device geometry of uniform thickness *d* ≪ *λ*, allowing us to model the geometry as a two-dimensional problem with a sheet supercurrent density and an effective penetration depth *λ*_eff_ = *λ*^2^/*d*. The geometry of the layer was then partitioned to perform Finite Element (FEM) simulations. The magnetic response was then obtained by setting a bias field and modeling the supercurrent density via the stream function^49^. The simulations in Fig. 2d used a rectangular island while cutting out a circular section of radius *r*_0_ at the gate-facing side of the island.

## Supplementary information

Supplementary Information

Transparent Peer Review file

## Data Availability

The data that support the findings of this study are available from the corresponding author upon request.
