# Supplementary material for: Imaging of gate-controlled suppression of superconductivity via the Meissner effect
Source: Nat Commun. 2026 Jul 18;17:8789. doi: 10.1038/s41467-026-75567-8 (PMC13493872; doi:10.1038/s41467-026-75567-8)
Supplement: Supplementary file 1 [file 41467_2026_75567_MOESM1_ESM.pdf]

Supplementary Information:
Imaging of Gate-Controlled Suppression of Superconductivity via the Meissner Effect

K. J. Knapp[1†], U. Ognjanovic[1†], P. J. Scheidegger[1†], L. Ruf[2],
S. Diesch[1], E. Scheer[2], A. Di Bernardo[2,3], and C. L. Degen[1,4*]

[1] *Department of Physics, ETH Zürich, Otto Stern Weg 1, 8093 Zürich, Switzerland.*

[2] *Department of Physics, University of Konstanz, Universitätsstrasse 10, 78464 Konstanz, Germany.*

[3] *Dipartimento di Fisica "E. R. Caianiello", Università degli Studi di Salerno, via Giovanni Paolo II 132, 84084 Fisciano, Salerno, Italy.*

[4] *Quantum Center, ETH Zürich, 8093 Zürich, Switzerland. and*

[†] *These authors contributed equally.*

(Dated: June 16, 2026)

SUPPLEMENTARY NOTE 1: DETERMINING THE QUENCHING RADIUS r_0

Approach 1: Fitting of line cuts

The first approach for determining r_0 involves the fitting of line cuts across the magnetic field maps by numerical simulations using the SuperScreen software package (Ref. [1] and Methods). As the line cut profiles are strongly affected by the presence of vortices, this method is ill-suited for images with significant flux penetration in the center of the superconducting island – namely those of devices D2a and D2b at low gate powers, and of device D3. Furthermore, the scan width in the D2a dataset is too narrow to support reliable fitting. The method was therefore only applied to the subset of D2b images that do not feature vortices.

The fitting procedure is illustrated in the figure below. From the $B_{\rm NV}$ field maps, where $B_{\rm NV}$ is the projection of the stray field along the NV symmetry axis (see Methods), we extract multiple horizontal line cuts through the center of the device, specifically across the gate region where the quenching is the largest. Next, these line cuts for each map are averaged to remove outliers and improve the quality of the dataset.

For the simulated field maps, we assumed a rectangular geometry for the Nb island with a fixed height and variable width w; the variable width accounts for the shrinking of the superconducting region due to quenching by the GCS. Fitting was performed using a custom routine adapted from [2] to account for common and shared fitting parameters across the dataset. Considering that the computationally expensive parts are the generation of (fine) meshes and the FEM simulation of the stream functions, we created a lookup table of FEM solutions for varying widths, penetration depths, and bias fields. This table was used

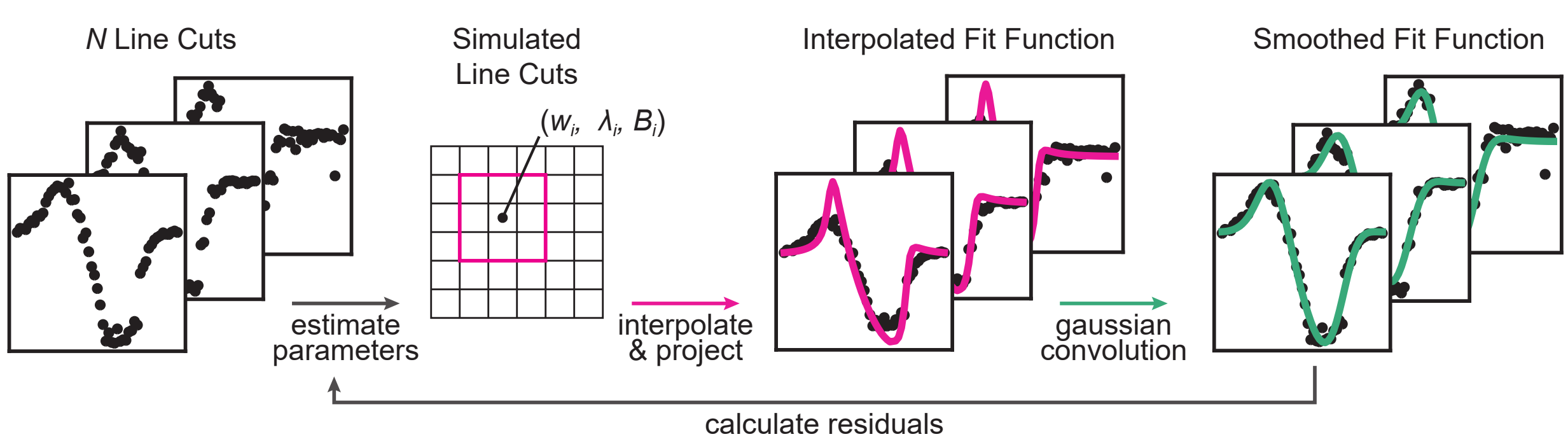

* degenc@ethz.ch

to interpolate for devices with parameters in-between the simulated values and to make projections onto the NV plane with different stand-off heights. The final magnetic field map was convolved with a Gaussian to account for broadening due to mechanical vibrations caused by the fridge's pulsed-tube coolers [3].

To extract the stand-off height z_0, London penetration depth λ, and device width w, we calculated the residuals for all line cuts while fixing common parameters like the stand-off height, NV angles, broadening, the penetration depth, and the horizontal reference point of the image. The quenching radius is then given by the relative change of the fitted width w compared to the original (unquenched) width w.

The r_0 vs. P data extracted by this method from device D2b is plotted in Fig. S3. Results for other fit parameters are collected in Table S2.

Approach 2: Locating extrema in B_x field maps

The contour of the superconducting region can also be estimated by locating characteristic features in the stray magnetic field at the boundary of the superconducting domain. For well-screened islands, the extrema in $B_x(x)$ and $B_z(x)$ align well with the superconducting domain edge, as illustrated in Fig. S4**a**. Crucially, this method does not rely on fitting a specific functional shape to the data and is also applicable in the presence of flux penetration in the interior of the island.

Due to qualitative differences between magnetometry images of devices D2a/D2b and D3, we developed separate algorithms to extract r_0 in each case. For D2a and D2b, we located the extrema in B_x magnetic field maps, where the edge signature is more pronounced compared to B_z. To improve the robustness, we applied Gaussian smoothing and averaged over multiple horizontal line-cuts across the device center. The quenching radius r_0 at a given gate power P was then determined by measuring the shift in the position of the minimum of the averaged $B_x(x)$ profile relative to its position at zero gate power. The results of this analysis are summarized in Fig. S4**b**. A fit to the 0.45 K dataset using Eq. (3) in the main text yields $r_d = 0.65\,\mu$m and $P_d = 0.36\,\mu$W. As shown in Fig. S4**c**, this dataset also exhibits a clearer independence of r_0 from the applied gate voltage V_{G} compared to the D3 dataset (see Fig. 3**b**).

The magnetometry images of the quenched region around gate (G1) of device D3 showed

two important qualitative differences compared to devices D2a and D2b. First, we found that the magnetic field was incompletely screened above the bulk of the Nb island (Fig. S5), probably due to a combination of vortices and Nb grain boundaries. This made it difficult to locate the extrema in B_x or B_z. Instead, the maximum of $|\partial B_x(x)/\partial x|$ provided the most reliable signature. Second, due to the increased size of the island, the shape of the imaged stray field around gate (G1) is not affected by boundary effects. We therefore determine the maximum in $|\partial B_x(x)/\partial x|$ for each row, yielding a curve that follows the domain edge over the full extent of the scan window. Again, we used outlier removal and Gaussian smoothing to improve the robustness.

The result of this procedure is shown in Fig. S4**d** for gate G1 of device D3. This curve clearly confirms the circular symmetry of the GCS quenching. Integrating over the curve and subtracting the integral over a reference curve acquired at zero gate power yields an area A_q that approximates the quenched region. However, because the curve follows the maximum in $|\partial B_x(x)/\partial x|$, it is systematically offset by an unknown distance d from the true domain edge. To find the quenching radius r_0, this offset must be considered. For a circularly quenched region of radius r_0 and a center located δ to the left of the device edge, the extracted area is

$$A_\mathrm{q}(r_0, \delta, d) = (r_0 + d)^2\theta - (r_0 + d)(d + \delta)\sin(\theta), \tag{S1}$$

for $r_0 \geq \delta$, where $\theta = \arccos\left(\frac{d+\delta}{r_0+d}\right)$. Although Eq. (S1) cannot be inverted analytically, r_0 can be extracted numerically if d and δ are known.

We estimate d by fitting circle segments to the extracted curves. To ensure that the fitting function accurately captures the shape of the extracted curves, we model them as the intersection between a circular domain and a fixed reference line (dashed black in Fig. S4**d**), which corresponds to the extracted curve at zero gate power. The circle radius and center position are treated as fit parameters. At high gate powers ($P \geq 2\,\mu$W), where the quenching region is large enough for reliable fitting, we simultaneously fit this model to multiple extracted contours, constraining the circle center to be shared across the dataset while allowing the radius to vary between scans. This procedure provides an estimate of the offset between the NEP injection site and the extracted contour at zero gate power, which is expected to be equal to $d + \delta$. Assuming $\delta = 35\,$nm, corresponding to half the distance of gate (G1) from the device edge, we get $d = 1.7\,\mu$m. An identical fitting routine was

employed to estimate the distance of hot spots for configurations where voltage was applied from (G4) to the island or between (G4) and (G5).

The relatively large systematic uncertainty in determining d was accounted for by assuming a large uncertainty of $\sigma_\mathrm{d} = 0.5\ \mu$m. Additionally, the uncertainty in the extracted area A_q arises primarily from shifts in the scan window with respect to the reference scans at zero gate power, which was accounted for by locating reference points and shifting the scan windows accordingly. A comparison of the enclosed area between zero power reference curves showed that the remaining error in A_q due to these shifts is limited to $\sigma_{A_\mathrm{q}} = 0.24\,\mu\mathrm{m}^2$. Both of these errors were propagated using a Monte Carlo simulation to determine the error bars of r_0 in Fig. 3**a**. In particular, we generated normal distributions of $X_d \sim N(d, \sigma_d)$ and $X_{A_\mathrm{q}} \sim N(A_\mathrm{q}, \sigma_{A_\mathrm{q}})$ from which we drew $N = 1000$ random samples $\{d^i, A_\mathrm{q}^i\}_i$. For each samplme pair, we calculate the corresponding quenching radius r_0^i by inverting equation S1. The error bars of r_0 correspond to the standard deviation of the resulting sets $\{r_0^i\}_i$.

SUPPLEMENTARY NOTE 2: DERIVATION OF THE HOT-SPOT MODEL

We developed a simple diffusion model to explain the power dependence of the quenching radius r_0 on the gate power P. We model the density of the non-equilibrium particles $\rho(\mathbf{r})$ by solving the steady-state three-dimensional diffusion equation,

$$D\nabla^2\rho(\mathbf{r}) - \frac{1}{\tau}\rho(\mathbf{r}) + Q\delta(\mathbf{r}) = 0 \ , \tag{S2}$$

where D is the diffusion coefficient, Q is the excitation rate, and τ is the thermal relaxation time. The term $Q\delta(\mathbf{r})$ models particle injection at the origin and $\frac{1}{\tau}\rho(\mathbf{r})$ accounts for thermal relaxation of the non-equilibrium particles. This equation is solved by

$$\rho(r) = \frac{Q}{4\pi D r}\exp\left(-\frac{r}{\sqrt{D\tau}}\right) \ , \tag{S3}$$

where $r = |\mathbf{r}|$.

Substituting the diffusion length $r_\mathrm{d} = \sqrt{D\tau}$ and assuming a proportional relationship between the gate power and particle injection rate, $Q = \alpha P$, we arrive at

$$\begin{aligned} \rho(r) &= \frac{\alpha P}{4\pi D r}\exp\left(-\frac{r}{r_\mathrm{d}}\right) \ , \\ &= \frac{\alpha P\tau}{4\pi r_\mathrm{d}^2 r}\exp\left(-\frac{r}{r_\mathrm{d}}\right) \ , \\ &= \rho_\mathrm{d}\frac{r_\mathrm{d}}{r}\exp\left(-\frac{r}{r_\mathrm{d}} + 1\right) \ , \end{aligned} \tag{S4}$$

where $\rho_\mathrm{d} = \rho(r_\mathrm{d}) = \alpha P\tau/4\pi r_\mathrm{d}^3 e$, or equivalently,

$$\exp\left(\frac{r}{r_\mathrm{d}}\right)\frac{r}{r_\mathrm{d}} = \frac{e\rho_\mathrm{d}}{\rho(r)} \ . \tag{S5}$$

Solving for the quenching radius r_0 with critical density $\rho_\mathrm{c} = \rho(r_0)$, above which superconductivity becomes suppressed, we find

$$\frac{r_0}{r_\mathrm{d}} = W_0\left(\frac{e\rho_\mathrm{d}}{\rho_\mathrm{c}}\right) \ , \tag{S6}$$

where W_0 is the principal branch of the Lambert W function. Eq. S6 can be expressed in terms of gate power P by introducing a critical Power P_d, at which the quenching radius r_0 is equal to the diffusion length r_d. Using Eq. S4 we find

$$\rho_\mathrm{c} = \frac{\alpha P_\mathrm{d}\tau}{4\pi r_\mathrm{d}^3 e} \ , \tag{S7}$$

and thus

$$\frac{\rho_{\mathrm{d}}}{\rho_{\mathrm{c}}} = \frac{P}{P_{\mathrm{d}}} \,, \tag{S8}$$

and finally

$$r_0 = r_{\mathrm{d}} W_0 \left(e \frac{P}{P_{\mathrm{d}}} \right) \,. \tag{S9}$$

Note that a more realistic model, where the particle density is contained in a semi-infinite volume $z \geq 0$, can be obtained using the mirror method to enforce the Neumann boundary condition $\frac{\partial \rho}{\partial z}\big|_{z=0} = 0$

$$\rho_{z\geq 0}(\mathbf{r}) = \rho(x, y, z) + \rho(x, y, -z), \tag{S10}$$

simply yielding $\rho_{z\geq 0}(\mathbf{r}) = 2\rho(|\mathbf{r}|)$ for $z \geq 0$. Eq. (S9) is also valid in this case.

SUPPLEMENTARY TABLES

Critical temperature T_c	Critical field $B_{c,2}$	Critical current I_c	Retrapping current I_R	Critical current density j_c	Retrapping current density j_R
6.0(3) K	> 200 mT	230(10) μA	144(8) μA	$1.88(21) \cdot 10^6$ Acm^{-2}	$1.18(13) \cdot 10^6$ Acm^{-2}

Table S1. **Superconducting properties of Nb film as measured on device D1.**

Data subset	x_0 (μm)	λ (nm)	B_0 (mT) (fixed)	z_0 (nm) (shared)	θ_{NV} (°) (fixed)	ϕ_{NV} (°) (fixed)	σ (nm) (shared)
0.45 K, $V_G > 0$	5.89(2)	236(10)	1.1	104(50)	53	5	340(40)
0.45 K, $V_G < 0$	5.55(2)	236(10)	1.1	104(50)	53	5	340(40)
5.8 K, $V_G > 0$	4.58(7)	412(30)	1.1	104(50)	53	5	340(40)
5.8 K, $V_G < 0$	4.14(3)	412(30)	1.1	104(50)	53	5	340(40)
3.5 K	4.18(3)	219(10)	1.1	104(50)	53	5	340(40)

Table S2. **Extracted values from the fitted B_{NV} line cuts.** x_0 is the position of the left device edge. λ is the London penetration depth. B_0 is the bias field applied in the out-of-plane direction. z_0 is the NV stand-off distance. θ_{NV} and ϕ_{NV} denote the orientation of the NV quantization axis (Methods). σ is a spatial broadening parameter. All fits were performed on data of device D2b. We note large uncertainties in the fit results for σ and z_0, which is due to their interdependence.

SUPPLEMENTARY FIGURES

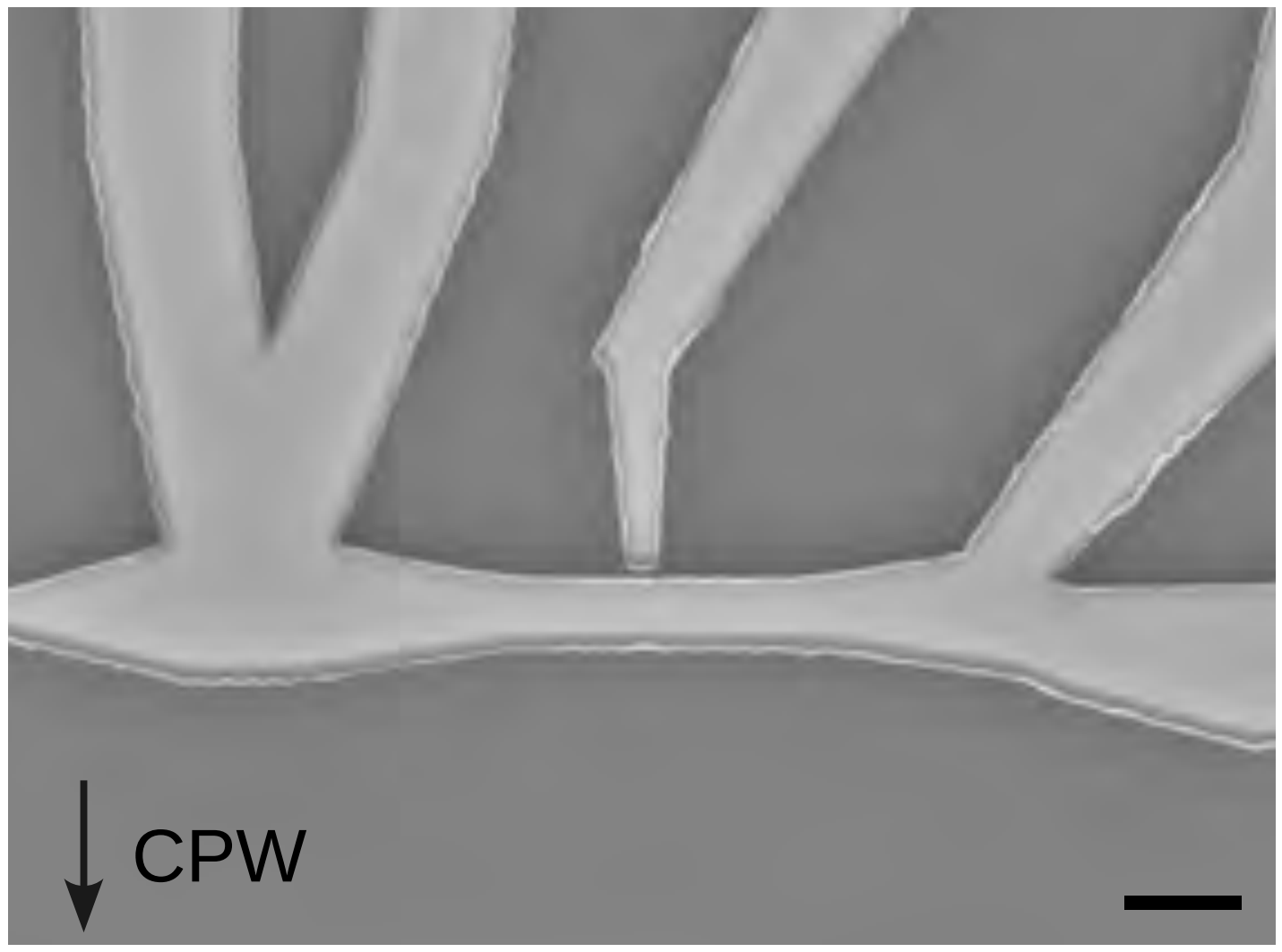

Figure S1. **SEM image of device D1.** Device D1 consists of a nanowire adjacent to a finger gate, resembling the device geometry typically used in transport-based GCS experiments. It is placed next to a coplanar waveguide (CPW) to drive the spin transition of the NV center. Due to the induction of eddy currents in the nanowire upon applying microwave drive pulses, the Rabi frequency of the spin transition became highly position-dependent, making it difficult to image the device [4]. The device was used to characterize the superconducting properties of the Nb film (see Table S1). Scale bar, 1 μm.

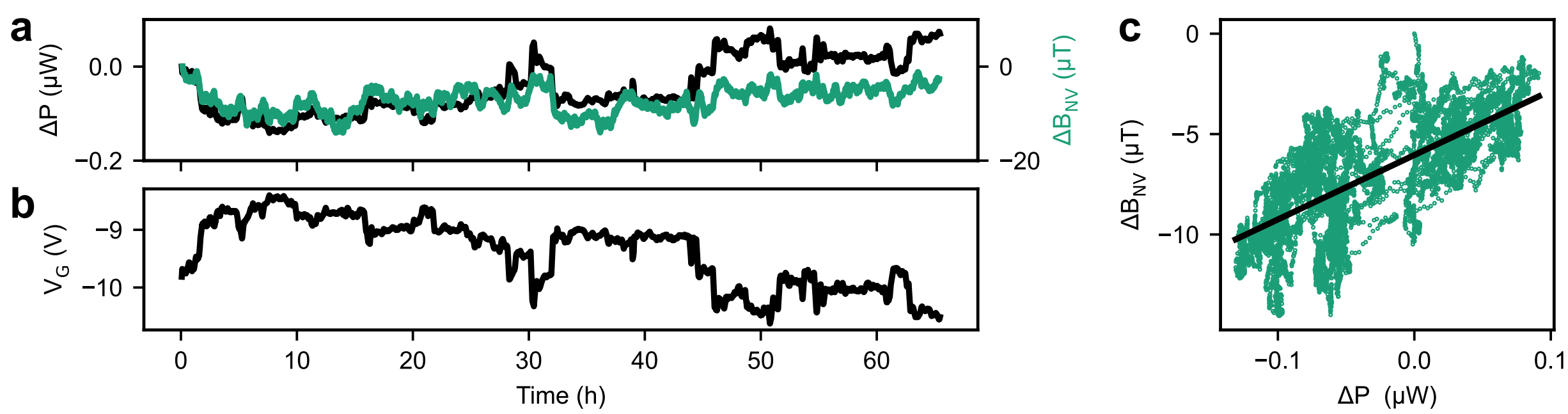

Figure S2. **Fluctuation of gate power and stray field over time.** **a**, Time traces of the gate power ΔP (black) and stray field ΔB_{NV} (green) measured at the right edge of the device (where the $\partial B_{\mathrm{NV}}/\partial x$ was highest), while applying $I_{\mathrm{L}} = 100\,\mathrm{nA}$ in constant current mode. The values are shown relative to their initial value at $t = 0$. The two curves are clearly correlated, indicating that the GCS follows P rather than I_{L}. Since the current is fixed, changes in P and B_{NV} are due to a change in voltage, ruling out that the extent of the quenching is purely determined by the current. **b**, Sourced gate voltage V_{G} during the experiment, while applying $I_{\mathrm{L}} = 100\,\mathrm{nA}$ in constant current mode. **c**, ΔB_{NV} directly plotted against ΔP including a linear trend line.

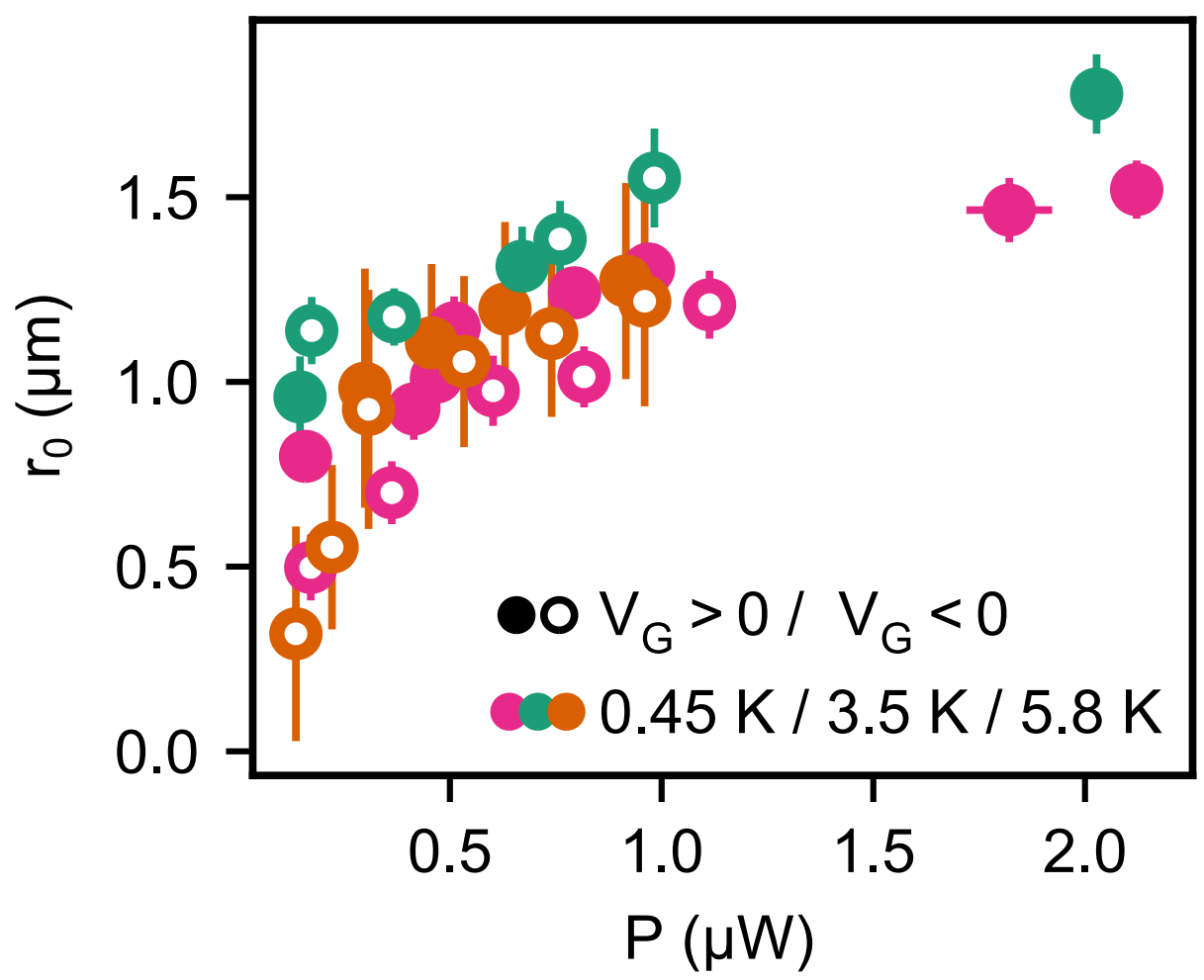

Figure S3. **The quenching radius r_0 obtained with line-cut fitting as a function of power P in device D2b.**

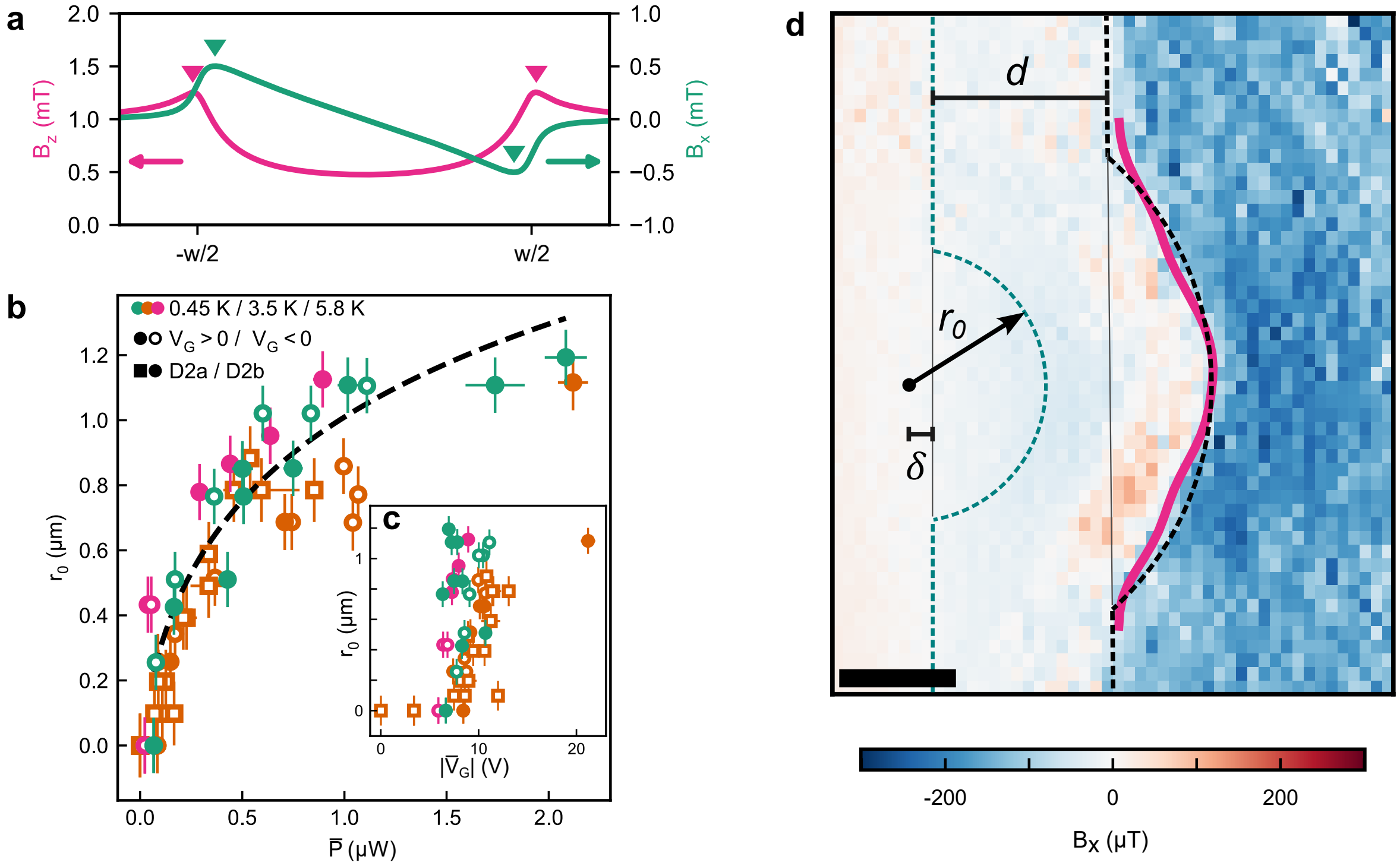

Figure S4. **Extrema-based extraction of the quenching radius** r_0. **a**, Simulated stray field components B_x (green) and B_z (pink) along a horizontal line cut through the center of a $w = 3\,\mu$m wide square superconducting island 150 nm from the sample surface. The field extrema (triangles) align with the edges of the superconducting domain. **b**, Quenching radius r_0 as a function of average gate power $\bar{P}$ for devices D2a and D2b, extracted by tracking the shift of the minimum in B_x across horizontal line-cuts. The dataset was acquired in constant current mode. **c**, Same dataset as in **b**, plotted as a function of the average gate voltage $|\bar{V}_G|$. **d**, Map of reconstructed B_x component around gate (G1) of device D3 at $P = 3\,\mu$W. The pink line traces the row-wise maxima in $\partial B_x/\partial x$, used to estimate the quenched area. The black dashed curve shows the result of a fit to the pink contour, obtained by modeling it as the intersection between a circular domain and a fixed vertical reference line corresponding to the extracted contour at zero gate power. Green dashed line marks the device edge, green dashed circle follows the inferred domain boundary. Scale bar, 1 μm.

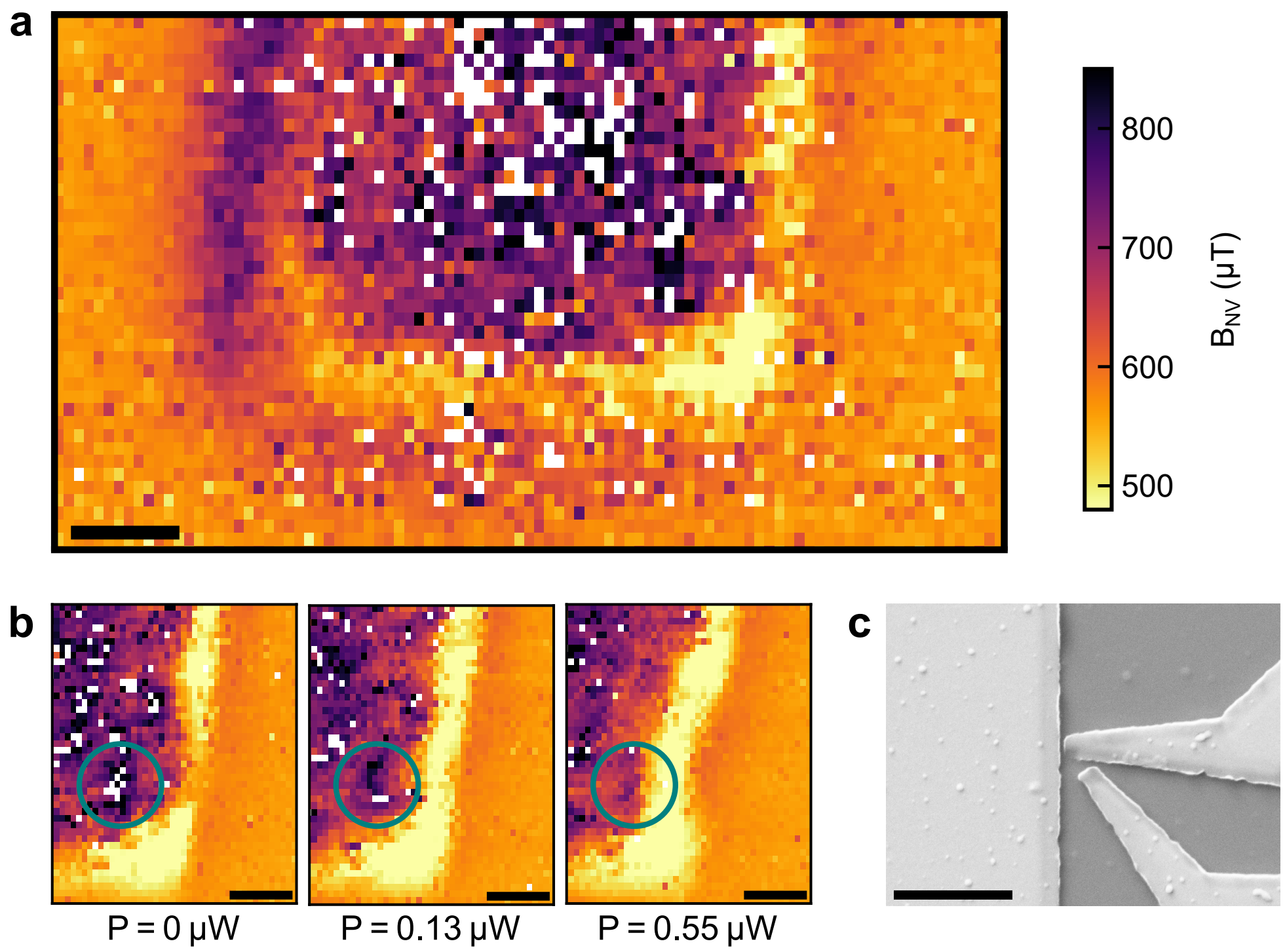

Figure S5. **Magnetic flux penetration of device D3.** **a**, Scan across the full width of the device revealing that the magnetic field is only Meissner screened at the edge of the device, while the center is penetrated by magnetic flux. **b**, B_{NV} maps zoomed in around gates (G4) and (G5) for increasing power applied between (G4) and the superconducting island. Spots (green circle) with particularly high field ($B_{\mathrm{NV}} \gtrsim 800\,\mu\mathrm{T}$) tend to remain fixed in position as the gate power is increased, suggesting that they are more strongly pinned to specific sites in the thin film compared to devices D2a and D2b. This difference in pinning behavior could arise from a higher density of nanoscale grains in D3 compared to D2a and D2b. Grain boundaries in polycrystalline thin films are known to act as strong pinning sites [5]. **c,** SEM image of gates (G4) and (G5), revealing nanoscale grains in the thin film. Scale bars, 1 μm.

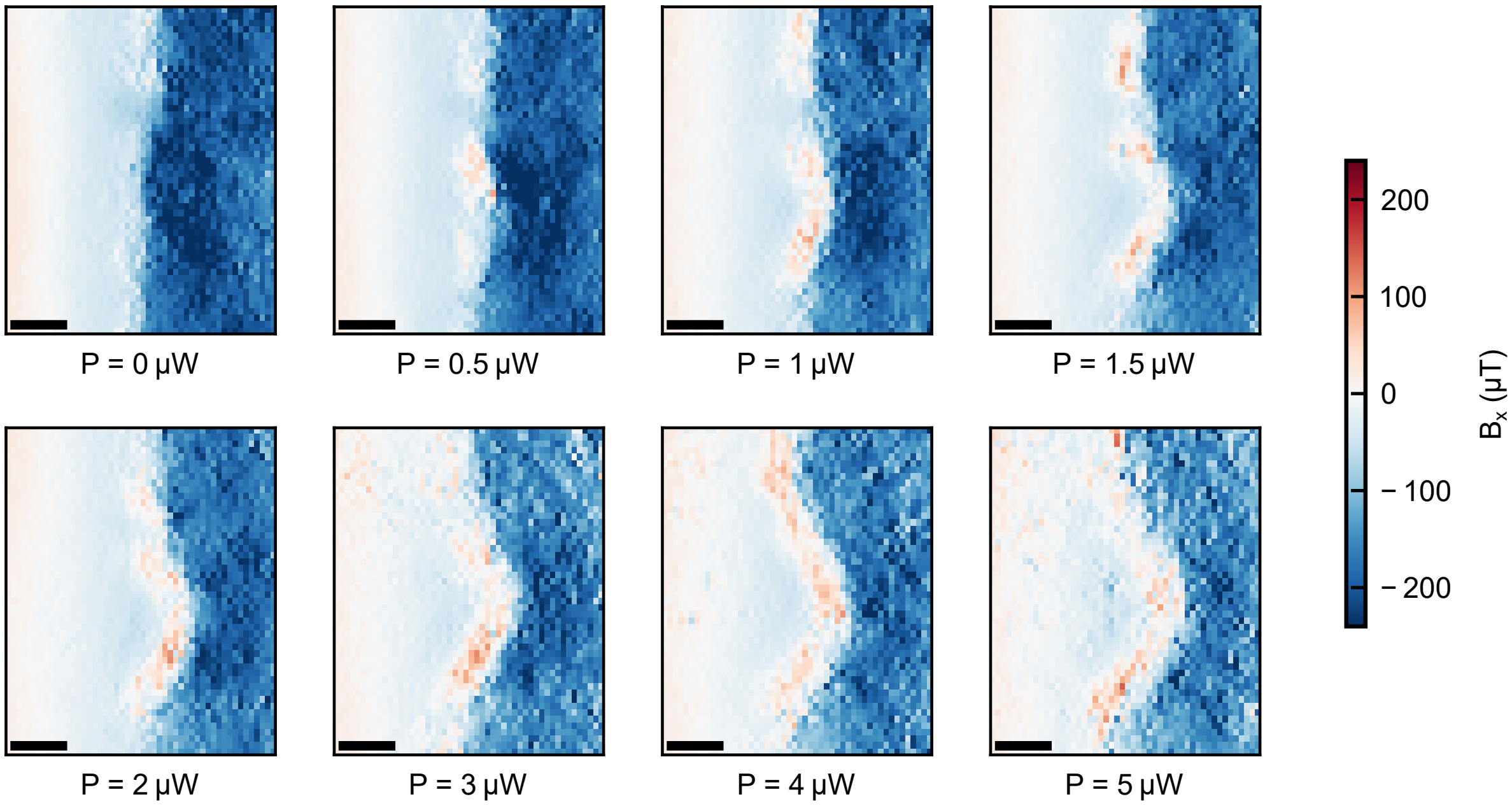

Figure S6. **Suppression of superconductivity around gate (G1) in device D3, demonstrating the spherical symmetry of the GCS effect.** Maps of B_x around gate (G1) of device D3 at varying gate powers. The suppression takes a circular shape across all gate powers.

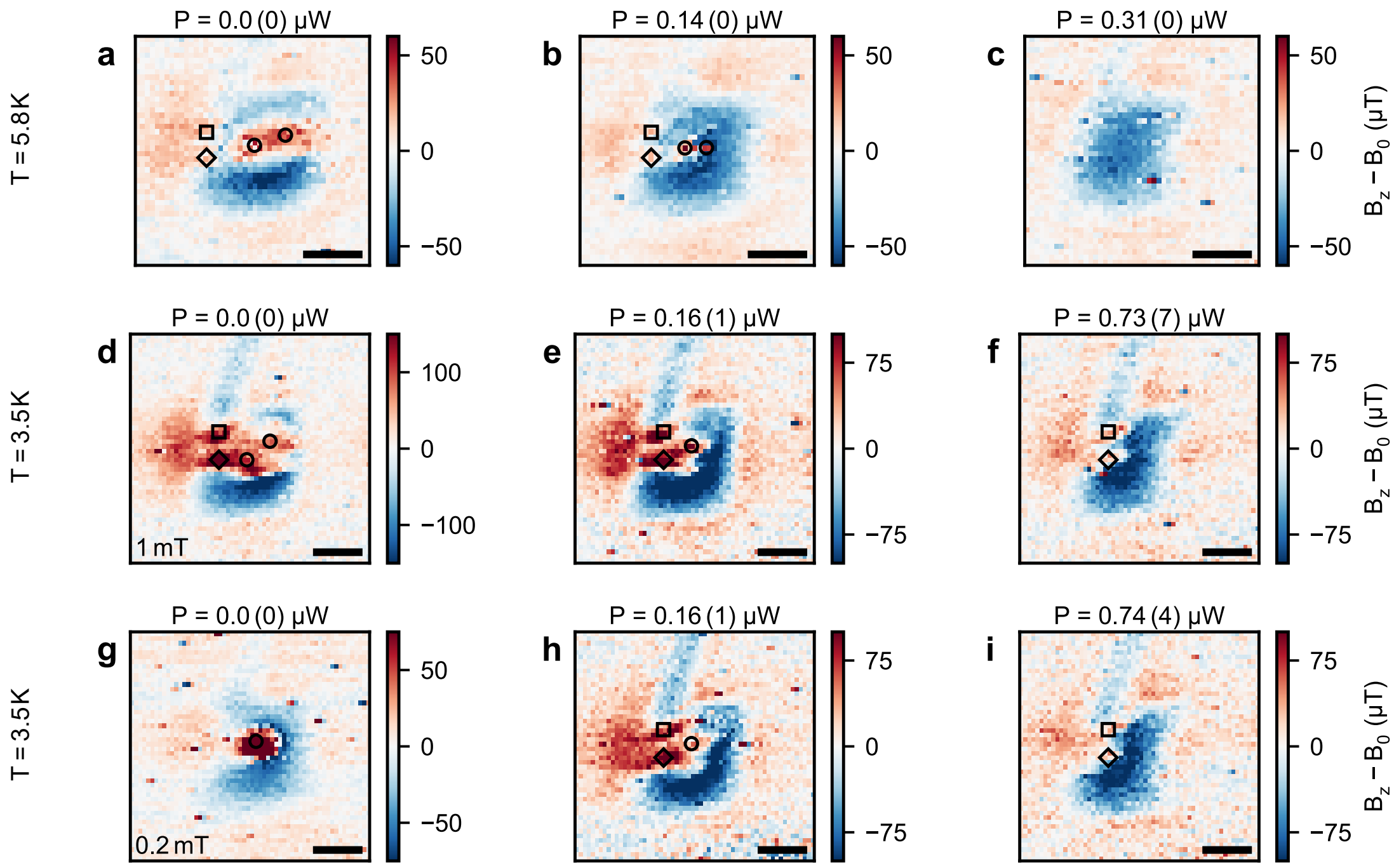

Figure S7. **Superconducting vortices in device D2b.** Images show the reconstructed out-of-plane magnetic field component B_z from scans acquired at 3.5 K and 5.8 K. All maps are shown with the applied bias field B_0 subtracted. Vortices are marked with symbols: circles denote shifting vortices, squares and diamonds mark persistent vortex locations across multiple scans and gate powers. At 3.5 K (**d–i**), vortices persist up to significantly higher gate powers compared to scans at 0.45 K (Fig. 2**c**) and 5.8 K (**a-c**). **a**, Vortex configuration at $T = 5.8\,\mathrm{K}$, $B_0 = 1\,\mathrm{mT}$ and $P = 0$, showing four vortices. **b-c**, Scans with $T = 5.8\,\mathrm{K}$, $B_0 = 1\,\mathrm{mT}$ and negative gate polarity. The vortices suddenly escape the microstructure for $P \gtrsim 0.2\,\mu\mathrm{W}$. **d**, Vortex configuration at $T = 3.5\,\mathrm{K}$, $B_0 = 1\,\mathrm{mT}$ and $P = 0$, showing four vortices. **g**, At lower field ($B_0 = 0.2\,\mathrm{mT}$), a single vortex remains. The number of vortices in **a,b** agrees well with the expected vorticity obtained by dividing the magnetic flux flowing through the sample area by the flux quantum $\phi_0 = h/(2e)$. **e-f**, Scans with $T = 3.5\,\mathrm{K}$, $B_0 = 1\,\mathrm{mT}$ and positive gate polarity. When increasingly higher power is applied to the gate, vortices leave the microstructure as the superconducting area gets increasingly quenched. The number of vortices is correlated with the effective superconducting area. This is in contrast to the datasets acquired at 0.45 K and 5.8 K, where all four vortices disappear at the same, low power. **h–i**, Scans with $T = 3.5\,\mathrm{K}$, $B_0 = 1\,\mathrm{mT}$ and negative gate polarity, showing that the vortex displacement and escape are not dependent on the gate polarity, and measurements at negative setpoints perfectly mirror the positive polarity data. Scale bars, 1 μm.

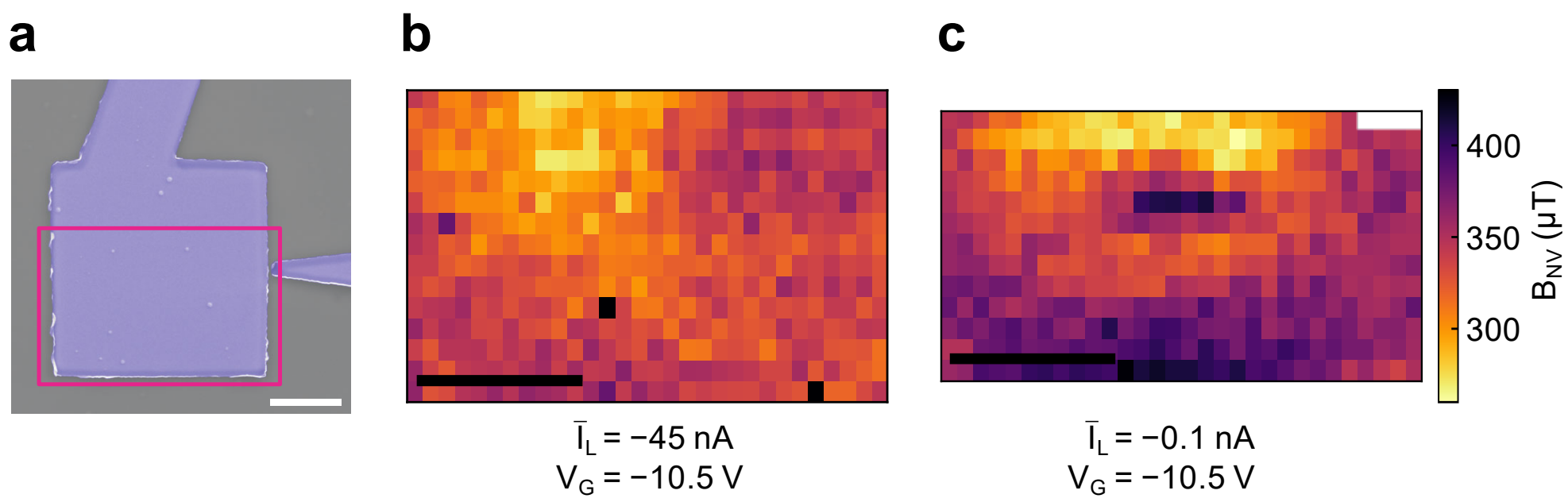

Figure S8. **Constant voltage measurements.** **a**, Scanned area of the device (D2a) indicated in a false-colored scanning electron micrograph. **b**, First measurement in which a suppression of the Meissner response can be observed with $V_G = -10.5\,\mathrm{V}$ applied. **c**, Subsequent measurement where no suppression is visible, although the same $V_G = -10.5\,\mathrm{V}$ is applied. The current-voltage relationship changed drastically, which is reflected in a significantly lower average leakage current (~ 450 times less). The scans **b-c** were performed at $B_0 = 1\,\mathrm{mT}$ and $T = 3.5\,\mathrm{K}$ in constant voltage mode. The scale bars indicate $1\,\mu\mathrm{m}$, while the colorbar applies for both **b-c**.

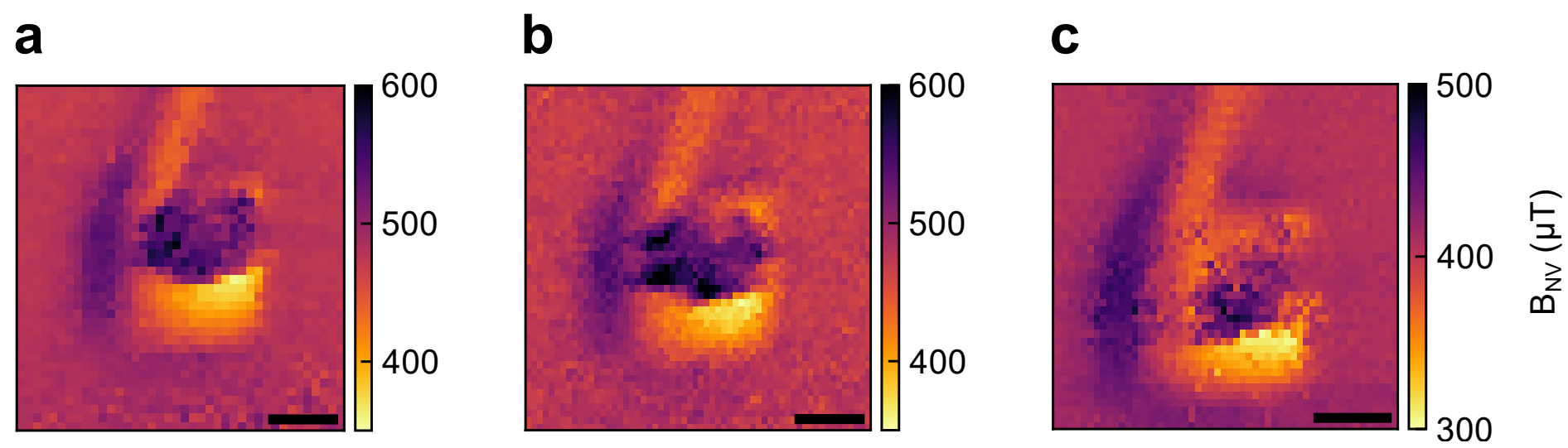

Figure S9. **Vortex pattern in device D2b after thermal cycling.** Images of $B_{\rm NV}$ at zero gate power $P = 0$, $B_0 = 1\,$mT and $T = 3.5\,$K. **a**, Vortex pattern immediately after cool down. **b**, Vortex pattern after having applied gate powers up to $P = 2.6\,\mu$W. **c**, Vortex pattern in a subsequent cooldown. The vortex positions have shifted compared to the configuration in **a**.

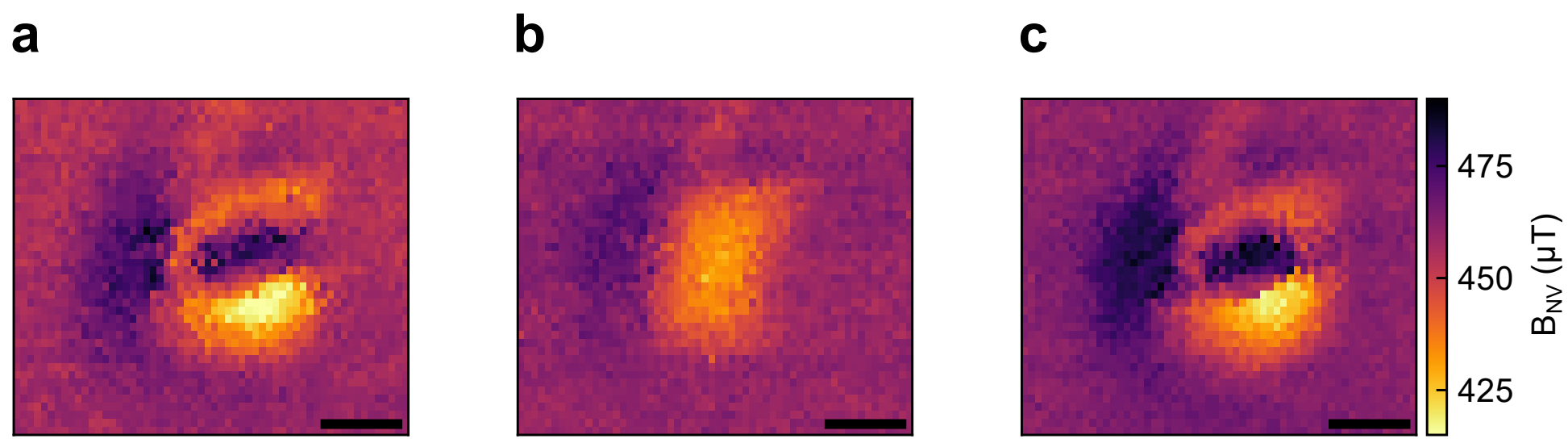

Figure S10. **Reproducibility of vortex position in device D2b within the same cooldown.** Images of B_{NV} acquired at $B_0 = 1\,\mathrm{mT}$ and $T = 5.8\,\mathrm{K}$. **a**, Vortex pattern at zero gate power ($P = 0\,\mu\mathrm{W}$). **b**, Subsequent scan with applied gate power $P = 0.54\,\mu\mathrm{W}$, showing the expulsion of the vortices from the microstructure. **c**, Final scan after returning to zero gate power. The vortex pattern recovers the configuration observed in **a**, demonstrating a reproducible pinning. Scale bars indicate 1 μm, the colorbar applies to all panels **a-c**.

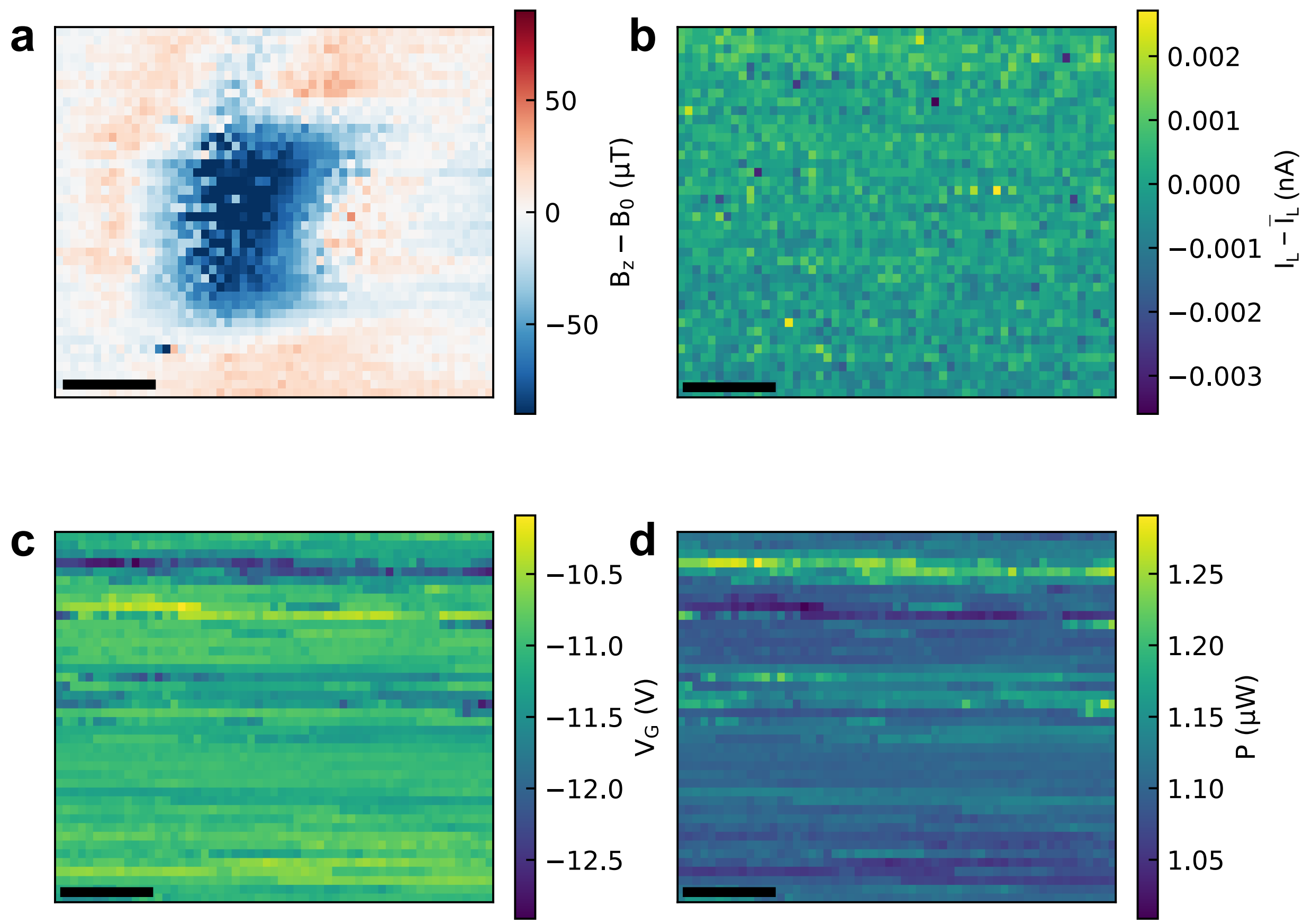

Figure S11. **I_{L}, V_{G} and P as function of scan position.** Maps of leakage current, gate voltage and power dissipated by leakage current measured at each pixel during a magnetometry scan of device D2b in constant current mode, acquired with $B_0 = 1\,\mathrm{mT}$ at $T = 3.5\,\mathrm{K}$. The scale bars are $1\,\mu$m. **a** Reconstructed magnetometry map of B_z, where we can observe quenching. **b** Corresponding map of leakage current, from which we have subtracted the average current $\bar{I}_{\mathrm{L}} = -100\,\mathrm{nA}$. **c** Corresponding map of V_{G}, which shows voltage variations approximately from $-10.5\,\mathrm{V}$ to $-12.5\,\mathrm{V}$. These variations do not have corresponding features in the reconstructed magnetometry map. **d** Map of P, which is of related to **b-c** by $P = I_{\mathrm{L}} V_{\mathrm{G}}$.

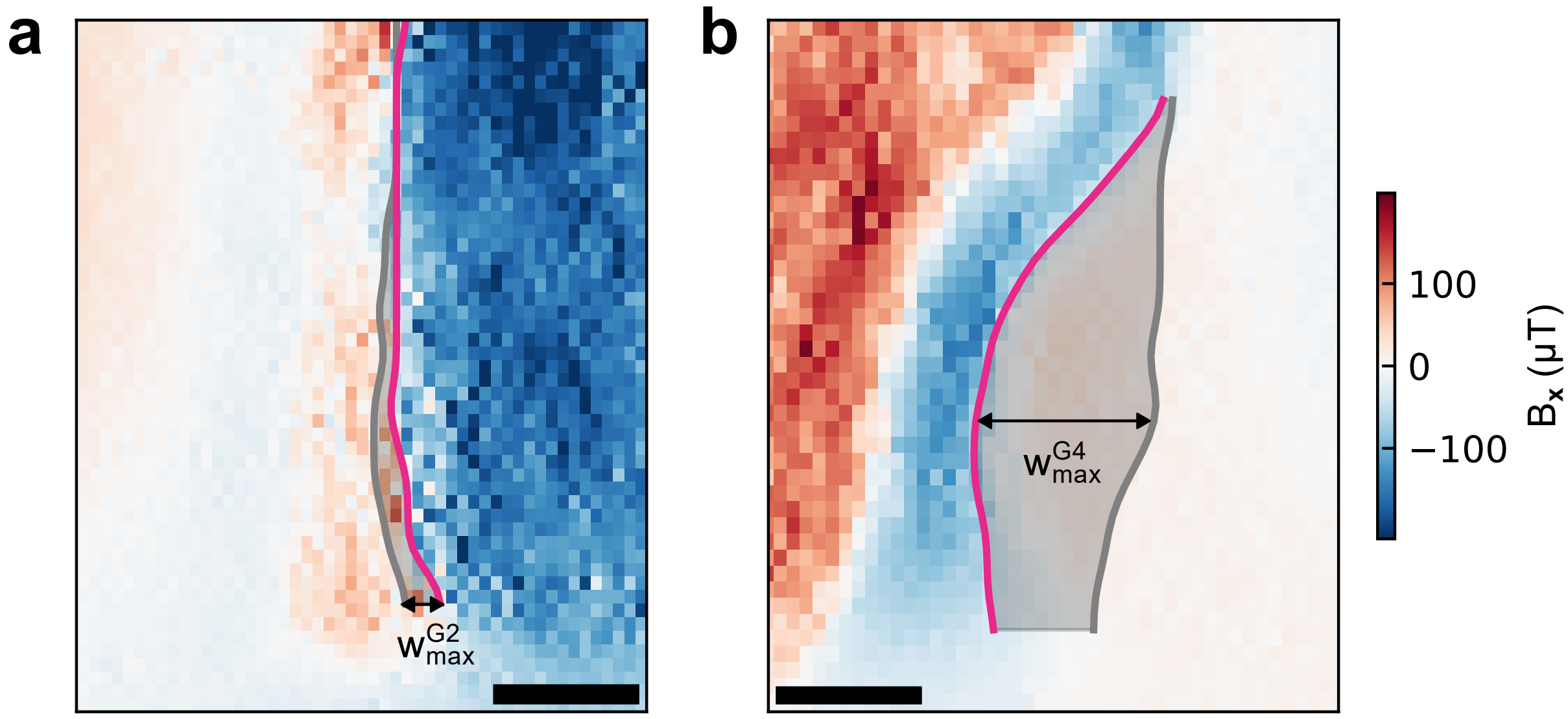

Figure S12. **Quantitative comparison of quenching extent for different gate geometries** Maps of the B_x stray field component around gates **a** G2 and **b** G4, acquired at gate power of $P = 2\,\mu$W. The pink lines trace the row-wise maxima of the spatial derivative $|\partial B_x/\partial x|$, marking the boundary of the superconducting domain as described in Section . The gray shaded areas indicate the spatial extent of the suppression relative to the zero-power reference (gray lines). The double-headed arrows indicate the maximum quenching width w_{max} perpendicular to the gate edge. Despite identical dissipated power, the suppression extends significantly further for the point-like injection ($w_{\mathrm{max}}^{\mathrm{G4}} = 1.23\,\mu$m) compared to the extended gate ($w_{\mathrm{max}}^{\mathrm{G2}} = 0.28\,\mu$m), suggesting that the local current density determines the quenching efficiency. Scale bars, $1\,\mu$m.

SUPPLEMENTARY REFERENCES

[1] L. B.-V. Horn and K. A. Moler, Superscreen: an open-source package for simulating the magnetic response of two-dimensional superconducting devices, Computer Physics Communications **280**, 108464 (2022).

[2] S. Ernst, Employing nitrogen-vacancy centers in diamond for scanning probe microscopy from cryogenic to room temperature, PhD Thesis, ETH Zurich (2024).

[3] P. J. Scheidegger, S. Diesch, M. L. Palm, and C. L. Degen, Scanning nitrogen-vacancy magnetometry down to 350 mk, Applied Physics Letters **120**, 224001 (2022).

[4] P. Scheidegger, Probing superconductivity with a milli-kelvin scanning nitrogen-vacancy magnetometer, PhD Thesis No. 30465, ETH Zurich (2024).

[5] S. M. Altanany, I. Zajcewa, T. Zajarniuk, A. Szewczyk, and M. Z. Cieplak, Vortex dynamics in disordered niobium thin films, Physical Review B **109**, 214504 (2024).
