# Supplementary material for: Imaging of gate-controlled suppression of superconductivity via the Meissner effect
Source: Nat Commun. 2026 Jul 18;17:8789. doi: 10.1038/s41467-026-75567-8 (PMC13493872; doi:10.1038/s41467-026-75567-8)
Supplement: Supplementary file 2 [file 41467_2026_75567_MOESM2_ESM.pdf]

nature portfolio

Peer Review File

Imaging of Gate-Controlled Suppression of Superconductivity via the Meissner Effect

Corresponding Author: Professor Christian Degen

This file contains all reviewer reports in order by version, followed by all author rebuttals in order by version.

Version 0:

Reviewer comments:

Reviewer #1

(Remarks to the Author)
The authors apply scanning NV magnetometry to study the gate control of supercurrent (GCS) effect in superconducting niobium. The signature of GCS is the suppression of superconductivity by a gate voltage applied near a superconductor. Here, they rely on the Meissner effect to study GCS via magnetic field map obtained with a scanning NV magnetometer. To quantify the strength of the gate modulation, they use total dissipated power as a proxy and report their results with power as the independent variable. The suppression of superconductivity is observed from the static field map around a Nb island with a nearby gate electrode at different applied power (Figure 2). The authors observe an enhanced suppression at higher applied power. Quantitatively, they extract a quenching radius from the field map as an indicator to argue that the quenching depends on the applied power, but not on temperature, electric field, and the polarity of the applied voltage (Figure 3a). They propose a hot-spot model assuming the quenching of superconductivity is caused by the injection of hot carriers that break Cooper pairs and then establish the quantitative relation between the quenching radius and applied power by modeling the hot-spot using a diffusion equation (Figure 3, SI section 2). The authors also qualitatively study the quenching from different gate geometries (Figure 4).
To the best of our knowledge this is the first work to report the study of the GCS effect with Meissner effect, whereas previous works used electronic transport as a probe. The main claim of this work is the observation of GCS via Meissner effect and the quantification of quenching through analysis of the static magnetic field maps, which allows them to rule out several possible mechanisms and propose a hot-spot diffusion model. The results are interesting and show how local magnetic imaging can provide new insights in superconductivity; we believe the manuscript will be of interest to both the condensed matter and quantum sensing communities. we would support publication in Nature Communications in broad terms, however we have several questions about the overall basic interpretation of the data:
1. The main claim of this work is centered around the hot-spot model, however the authors never explain how the carrier injection happens in the first place. The Nb island and the gate are separated by 80 nm, and the whole metal stack is deposited on top of an insulator (SiO_2). Thus, it is unclear how the carriers (in this case, electrons) could enter the insulator and then diffuse from one electrode through the insulator to the other one.
2. A possibly related question is: how perturbative is the NV measurement? The authors are constantly shining green light on the sample, which could assist in carrier injection. Presumably the measured magnetic field is from phase evolution in the dark, but have the authors measured long term effects after laser illumination? For example, if there are long-lived trapped charges in the SiO_2 insulating layer, that could have profound effects on carrier transport / carrier injection.
3. What is the pattern and time scale of the change in the turn-on voltage? How often does this voltage change? And how long does it take to obtain a magnetic field map? In figure 3b, if you extrapolate both datasets to $r0 = 0$, then you could define a reasonable turn-on voltage in this case (x-intercept) at the two different temperatures. Since it is known that it is time-consuming to obtain a magnetic field scan, I am assuming the time scale of the whole dataset should be on the order of days if not weeks. How could the extrapolated turn-on voltage be so stable over such a long time, which seems to contradicts what the authors report in figure 1c for the fluctuating turn-on voltage measured with electronic transport.
4. Following the previous point, the authors' interpretation to the data in figure 3b is confusing. The authors claim that they do not see a correlation between gate voltage and their extracted quenching radius, thus ruling out the possibility of direct electric field effect. However, it is pretty probable from figure 3b that the relation between gate voltage and r0 could be linear. In addition, the fact that you could obtain a well-defined turn-on voltage that is stable over all experiments based on the x-intercept for a linear fit to each dataset probably suggests a minimum electrical field for GCS, thus contradicting the authors' claim that they rule out electric field as a source of the quench. Moreover, figure 3b shows that the turn-on voltage is lower at lower temperature, suggesting that temperature also plays some role here. The model that the authors present provides a fit

to the data in figure 3a, yet the conclusion that the authors draw from this model seems to disagree with what figure 3b tells. The authors should address this discrepancy in detail to justify their claim that GCS is independent of electric field and temperature.
5. The authors show data in figure 4 for different gate configurations. The authors should consider carry out the same quantitative analysis for at least one more different gate geometry, for example, G2/3. If the source of the hot carriers is correlated with the point with the highest current density as the authors claim, then that should show up as a much lower \alpha in their model for G2/3 since the electrode geometry is more spread out. The author should provide this additional dataset to support their claim, and explain how they expect the parameter \alpha to scale with respect to other factors such as electric field intensity or current density. That being said, the author should address point 2 and 3 first to justify the main claim of this work.
6. Could the authors please explain how they identify that there are four superconducting vortices in figure 2c? I do not think I know how to read from that map that there are vortices and their positions. Also, would the vortices reappear at the same positions if the sample is thermal-cycled (heated above critical temperature and cooled down again)?

Reviewer #2

(Remarks to the Author)
The manuscript is focused on imaging the stray magnet field properties of thin film superconducting niobium under the influence of a local gate electrode using nitrogen-vacancy center magnetometry at sub-kelvin temperatures.

The authors argue that they are able to determine the origin of “gate controlled superconductivity” (GCS) via measurements of the Meissner effect in regions of the superconducting film that are near a local gate. They are argue that the GCS effect results from current leakage between the gate and the superconductor, which injects hot quasiparticles that then quench superconductivity in a circular region whose radius reflects a diffusion length.

These are an impressive series of experiments using scanning NV magnetometry at sub-kelvin temperatures, which provide direct imaging of superconducting characteristics and strong support for the hot quasiparticle injection mechanism of GCS. These measurements also provide quantitative insight into GCS that is largely inaccessible via conventional transport measurements. As such, I believe that the results could be broadly impactful to mesoscopic superconductivity and superconducting technologies. Before recommending for publication, I believe the authors should address a few outstanding issues identified below.

1. The effect of the laser, required for optical readout of the nitrogen-vacancy center, is not discussed. This is an important limitation of NV center magnetometry that needs to be explicitly addressed particularly in this study.

The underlying mechanism for hot quasiparticle injection and complete quenching of superconductivity via gate leakage appears to depend on power dissipated through the gap between superconducting islands (~ 1 microwatt or less in a ~100nm squared area associated with the gap between superconducting islands). To my knowledge NV center readout requires up to two orders of magnitude higher laser power (probably around 100 microwatts), which in this scanning experiment is focused tightly onto the measurement location via the scanning probe pillar presumably something like 100 nm in diameter. The power density associated with the laser therefore appears to far exceed that associated with the gate leakage. I am aware that the mechanism of power absorption is different in the case of gate leakage versus optical illumination. However, by this argument of power density, I would think that the laser would have a huge effect on the stray field data as it moves around the superconducting film during a tip raster scan – locally creating its own hot spot of non-equilibrium quasiparticles underneath the measurement region.

This effect could be relevant to the observed suppression of Meissner screening in the bulk film in Fig. 4. The laser may also drag around vortices in cases where they are present like in Fig. 2A. I am also wondering to what extent the laser extrinsically “expands” the radius of quenched superconductivity around the gate by adding an additional source of non-equilibrium quasiparticles, which would affect the quantitative analysis around Fig. 3.

The authors should state the laser power used, the width of the diamond pillar, whether it is a continuous wave or pulsed measurement, and include some arguments about the extent they believe the laser is or is not affecting the results, as well as any steps they took to mitigate or understand the possible invasive effect of the laser. It could be a supplementary section that is referenced in the main text.

2. In reading the main text and supplement, it is very difficult to find out how exactly the values of r0 and their error bars are obtained in Fig. 3. This appears to be a critical piece of analysis in the present manuscript, but the text describing these methods in both the main text and supplement are confusing.

a. There are two separate analysis methods brought up in the main text when only one is used to obtain the values of r0 presented in Fig. 3. The main text is also unclear about exactly which method is used to obtain the presented r0 values. It seems that “approach 2: finding extrema” is the one used for Fig. 3, but this is not at all clear from reading the main text.

b. The description of the relevant “approach 2” in the main text is too vague “The second approach involves locating the field step in a Bx map and determining the shift relative to a reference map measured with VG = 0”. What is the shift they are referring to? What is a field step?

Can the authors state, in the main text, the exact analysis procedure that is used on device D3, list the fit parameters involved in this analysis, and state explicitly which parameters are are kept fixed as the gate power is varied? It could be useful to incorporate some version of Fig. S4D into Fig. 3. It is only after seeing Fig. S4D that I could understand what the actual free parameters are in this procedure and how r0 is reflected in the raw data.

c. The error bars in r0 in “approach 2” are obtained via Monte Carlo simulation according to the supplement, but no further information is provided. This needs to be explained in more rigorous detail.

3. “Curiously, near zero gate power, we observe a cluster of four superconducting vortices within the Nb island”

Can the authors describe in more detail, or label on the figure what features they are associating with four vortices? This is done in the supplement, and it would be useful to do the same in the main text.

6. Overall, I find it hard to discern what is actually the independent variable in many of the data shown in the manuscript.

a. The methods state that transport data in Fig. 1 are taken in “constant voltage” mode, whereas the caption of Fig. 1 states that it is taken in “constant current” mode.

b. The methods also state that magnetometry measurements on D3 are performed with the SMU in constant current mode, where the current is dynamically adjusted to keep the power fixed. Other magnetometry data is taken in standard constant current mode.

Throughout the manuscript, however, the scans are labeled or referred to by gate voltage “VG” (e.g. in Fig. 3b, and the surrounding text, as well as in related supplemental figures). Based on the methods, however, VG is never the independent variable in the magnetometry scans. It is either I_L or power, depending on whether the authors used the PID loop to keep power fixed or not. The figures and text should accurately reflect the independent variable which is controlled in the experiment.

4. How are the points labeled by VG in Fig. 3b? It appears to be the same data as in Fig. 3a, but replotted with a different x-axis. If power is being held fixed during each scan, VG could be changing by up to several volts during this procedure (I am inferring this from the transport data in Fig. 1). Therefore each r0 measurement is not associated with a single value of VG. Does the x-axis of Fig. 3b represent some “average” or “nominal” gate voltage over the course of a scan? If so, there should be an indication on the plot of the spread in VG for each data point.

5. “Finally, a direct electric field effect is also excluded because the quenching is not correlated with the gate voltage (Fig. 3b)”

The quenching does appear to be correlated with VG. Do the authors mean that they are not linearly correlated, and this excludes direct electric field effects? I would contest this given point 4 above – each extracted r0 data point does not have a single VG associated with it, so how can the authors conclude how r0 scales with VG?

7. The thermal diffusivity constant quoted for glass appears to be a room temperature value if I am not mistaken. This is probably not quantitatively relevant to the present experiment.

8. “For quasiparticle diffusion, D $\sim$ 5·10−4 m2/s”
It should be stated explicitly that this value corresponds to quasiparticles in niobium thin films as inferred from transport measurements.

9. Throughout the manuscript the authors refer to a London penetration depth. The length constant obtained via the stray field should be a Pearl length relevant for thin film superconductors. Are they converting the Pearl length to London penetration depth? If so, they should state how this is done.

Reviewer #3

(Remarks to the Author)
The paper by Scheidegger et al. reports imaging of Nb islands subjected to a large leakage current via scanning NV magnetometer. Various observations are reported on how the leakage current affects the superconducting state.

In my opinion, the paper shows limited amount of novelties for a Nature journal. The main novelty is that a leakage current suppressed Meissner screening. However, this is not extensively characterized, and it is based only on one thin film (Nb, 20 nm). Instead of simply showcasing the observation, the authors should understand it better, and in different films. If further understanding would be gained about this point I would reconsider my opinion.

Other key conclusions are all well-known facts. It is known that power has most effect (if voltage is sufficiently large). It is known that the impact on superconductivity rapidly decreases with distance. It is also known that the effect takes places via generation of a hot spot and spreading of phonons. I do not see significant breakthroughs with respect to these known facts.

Questions and comments

What happens to the vortexes after the voltage is set back to zero? Do they appear again, or they are gone forever?

The paper starts with a fake news: "It was recently discovered that supercurrents flowing through thin superconducting nanowires can be quenched by a gate voltage." In fact, a very strong and large body of work indicates that the gate voltage has no effect on superconductivity in metals.

The acronym GCS is wrong, and I suggest to stop using it. Tt is not gate controlled, and what is controlled is the switching current, not the supercurrent.

Second line of the paper: "owing to the possible new physics ". New physics does not mean anything.

Another wrong fact appears right afterwards: "A defining
feature of the GCS is the complete switching of a typically small superconducting volume into its normal state under the application of a sufficiently large gate voltage." Of course the gate voltage is not doing anything here. Second, I am not aware of evidence that reports the complete switching of superconductivity. In fact most reports show that the switching current goes to zero, not that superconductivity is quenched. I am concerned by the fact that the authors spread this unsupported information as "defining fact".

Finally "Although significant work has been put into developing a microscopic understanding of the GCS effect, its precise origin is still under debate." This is completely false, and it is likely written to make the paper more appealing. Everybody knows the origin of such effect since 2021-2022. There is no doubt in the community that there is no electric field involved.

Reviewer #4

(Remarks to the Author)
I co-reviewed this manuscript with one of the reviewers who provided the listed reports. This is part of the Nature Communications initiative to facilitate training in peer review and to provide appropriate recognition for Early Career Researchers who co-review manuscripts.

Version 1:

Reviewer comments:

Reviewer #1

(Remarks to the Author)
The authors have made several changes to the manuscript that address most of our questions; in particular their argument about phonon diffusion leading to Cooper pair breaking is reasonable. I would suggest, however, that their assertion that they cannot distinguish whether the diffusion length is related to phonon propagation or quasiparticle diffusion merits some more quantitative attention. Since their model assumes phonon propagation in the substrate, the authors should be able to give an estimate on the characteristic length of phonon propagation/dissipation given that the substrate is SiO_2/Si stack, whose properties are well-known. We believe they could also easily estimate the diffusion of quasiparticles in Nb, whose lifetime is probably a well-known quantity as the authors cited in their response to the next question. Thus, we would encourage the authors to provide an estimate of the relevant length scales and compare that to their measurement.

A more serious remaining concern: regarding our questions about the claimed lack of correlation between gate voltage and suppression of superconductivity, we still find the authors' explanations confusing. If the proposed mechanism arises from leakage current -> phonons -> quasiparticles, then shouldn't the same gate voltage give the same leakage current, and thus give reproducible suppression (comparing S4c to S8)? More to the point, there should just be a clear quantitative relationship, as well as an empirical I-V curve, that relates leakage current to applied voltage, and predicts the correlation between r and V_g.

Reviewer #3

(Remarks to the Author)
The paper improved significantly in the wordings, and I thank the authors for reflecting on my comments. When the authors claim "suppression of superconductivity", it is unclear if they mean that screening is absent, the sample turned resistive, or it turned normal. I still believe that GCS is the wrong wording, but I will not complain further on this point.

I find the novelty of this work still limited, as most of their findings were already well known. The observation that injection of high energy electrons suppresses screening is new and interesting. Overall, I favor publication.

Reviewer #4

(Remarks to the Author)
I co-reviewed this manuscript with one of the reviewers who provided the listed reports. This is part of the Nature Communications initiative to facilitate training in peer review and to provide appropriate recognition for Early Career Researchers who co-review manuscripts.

Version 2:

Reviewer comments:

Reviewer #1

(Remarks to the Author)
I appreciate the authors' added quantitative estimations of the quasiparticle and phonon diffusion lengths. They have addressed all remaining questions with the update, and I support publication.

Reviewer #3

(Remarks to the Author)
I think the paper is ready to be accepted.

Reviewer #4

(Remarks to the Author)
I co-reviewed this manuscript with one of the reviewers who provided the listed reports. This is part of the Nature Communications initiative to facilitate training in peer review and to provide appropriate recognition for Early Career Researchers who co-review manuscripts.

Open Access This Peer Review File is licensed under a Creative Commons Attribution 4.0 International License, which permits use, sharing, adaptation, distribution and reproduction in any medium or format, as long as you give appropriate credit to the original author(s) and the source, provide a link to the Creative Commons license, and indicate if changes were made.
In cases where reviewers are anonymous, credit should be given to 'Anonymous Referee' and the source.
The images or other third party material in this Peer Review File are included in the article’s Creative Commons license, unless indicated otherwise in a credit line to the material. If material is not included in the article’s Creative Commons license and your intended use is not permitted by statutory regulation or exceeds the permitted use, you will need to obtain permission directly from the copyright holder.
To view a copy of this license, visit https://creativecommons.org/licenses/by/4.0/

****

Editorial comment:

In addition to the reviewer reports below, we received a comment from a peer who was invited to review the manuscript, but did not have time to write a full report. The comment is: "(the authors claim) that "electron injection through vacuum is ruled out because the effect does not depend on gate polarity". This is a misunderstanding of non-equilibrium superconductivity. Injection of holes is fully equivalent to injection of electrons... in the reviewing process of the paper, please make sure that this wrong statement is corrected." In your point-by-point-response, please respond to this comment also, and we will ask the other reviewers to assess your response.

Authors: The voltages applied in our study correspond to many eV, which is far beyond the superconducting gap of a few meV. Therefore, the injection of electrons into vacuum is governed by Fowler-Nordheim field emission, which is polarity dependent considering the geometric asymmetry of the gates. Electrons are only emitted when the gate electrode is the cathode.

In response, we clarified the sentence in the main text: *"Second, electron injection through vacuum is ruled out because at the high gate voltages applied in our study and given the asymmetric gate geometry, this mechanism would depend on gate polarity."*

****

Reviewer #1 (Remarks to the Author):

The authors apply scanning NV magnetometry to study the gate control of supercurrent (GCS) effect in superconducting niobium. The signature of GCS is the suppression of superconductivity by a gate voltage applied near a superconductor. Here, they rely on the Meissner effect to study GCS via magnetic field map obtained with a scanning NV magnetometer. To quantify the strength of the gate modulation, they use total dissipated power as a proxy and report their results with power as the independent variable. The suppression of superconductivity is observed from the static field map around a Nb island with a nearby gate electrode at different applied power (Figure 2). The authors observe an enhanced suppression at higher applied power. Quantitatively, they extract a quenching radius from the field map as an indicator to argue that the quenching depends on the applied power, but not on temperature, electric field, and the polarity of the applied voltage (Figure 3a). They propose a hot-spot model assuming the quenching of superconductivity is caused by the injection of hot carriers that break Cooper pairs and then establish the quantitative relation between the quenching radius and applied power by modeling the hot-spot using a diffusion equation (Figure 3, SI section 2). The authors also qualitatively study the quenching from different gate geometries (Figure 4).

To the best of our knowledge this is the first work to report the study of the GCS effect with Meissner effect, whereas previous works used electronic transport as a probe. The main claim of this work is the observation of GCS via Meissner effect and the quantification of quenching through analysis of the static magnetic field maps, which allows them to rule out several possible mechanisms and propose a hot-spot diffusion model. The results are interesting and show how local magnetic imaging can provide new insights in superconductivity; we believe the manuscript will be of interest to both the condensed matter and quantum sensing communities. we would support publication in Nature

Communications in broad terms, however we have several questions about the overall basic interpretation of the data:

Authors: We thank the Reviewer for taking their time to assess our manuscript.

1. The main claim of this work is centered around the hot-spot model, however the authors never explain how the carrier injection happens in the first place. The Nb island and the gate are separated by 80 nm, and the whole metal stack is deposited on top of an insulator (SiO2). Thus, it is unclear how the carriers (in this case, electrons) could enter the insulator and then diffuse from one electrode through the insulator to the other one.

Authors: We thank Reviewer #1 for this important remark.

The suggested microscopic mechanism is sketched in Fig. 3c: a minute leakage current dissipates energy at the gate apex, generating high-energy non-equilibrium particles (NEPs). Since the current density is highest at the tip of the gate, this position acts at the hot spot. While the primary NEPs at the hot spot may include both electrons and phonons, the 80 nm SiO_2 layer acts as a barrier to direct electron injection. Therefore, we hypothesize that energy is primarily transmitted to the Nb island via phonons. Once in the island, these phonons may excite a high density of quasiparticles which diffuse laterally, locally suppressing superconductivity. As we are only able to record the spatial response of the Meissner screening, we cannot definitively distinguish whether the extracted diffusion length r_d is predominantly governed by the propagation of phonons within the substrate or the diffusion of quasiparticles within the superconducting film. Consequently, we identify r_0 and r_d as the characteristic length scales of the lateral spread of NEPs, which provides a quantitative spatial bound for the GCS effect regardless of the specific microscopic carrier.

Recent experiments [6, 20] suggest that the hot spot might also be at some charge traps in the gap between the gate and the island. However, within the spatial resolution of our experiment we cannot distinguish between these two scenarios.

To make this model even clearer, we rephrased our section "hot-spot model" (right column on page 3) as follows:

"*In the following, we argue that the quenching of superconductivity is caused by non-equilibrium particles (NEPs)—namely, hot electrons or phonons* [6, 13, 16, 17, 20]*, generated by the leakage current. The suggested microscopic mechanism is sketched in Fig*. 3c*: the NEPs, generated at the location of highest leakage current density (i.e., below the gate apex), form a local hot spot. This hot spot is similar to that observed in superconducting single photon detectors, however, it is not caused by heating [26]. The number of excited NEPs can exceed the number of charge carriers of the leakage current by far [14, 26]. Because of the relatively large distance between gate and Nb island, we argue that the NEPs reaching the island are mainly phonons. When impinging on the superconductor, the NEPs excite quasiparticles by breaking up Cooper pairs, leading to a suppression of superconductivity. While diffusing, both types of particles—phonons in the substrate and quasiparticles in the superconductor—also undergo thermal relaxation, eventually limiting the radius of the suppression.*"

2. A possibly related question is: how perturbative is the NV measurement? The authors are constantly shining green light on the sample, which could assist in carrier injection. Presumably the measured magnetic field is from phase evolution in the dark, but have the authors measured long

term effects after laser illumination? For example, if there are long-lived trapped charges in the SiO_2 insulating layer, that could have profound effects on carrier transport / carrier injection.

Authors: We thank the Reviewer for raising this question, which was also raised by Reviewer #2 (point 1). Both concerns are addressed here.

To address the reviewer's concern regarding laser-assisted carrier injection, we added Supplementary Figure S11, which maps the gate voltage, power, and leakage current at every scan position during an exemplary magnetometry scan. Since the transport quantities are uncorrelated to the device geometry, we conclude that the laser has no position-dependent effect on the transport properties.

Further, as pointed out by the Reviewer, in the pulse ODMR scheme, phase evolution and laser illumination are separated by ~1 us, which is much longer than the excitation and relaxation times of quasiparticles (<1 ns) [see new Ref. 48: Johnson, PRL, 1991]. Therefore, direct excitation of charge carriers during the magnetic measurement is unlikely.

Finally, as Meissner screening of the full device has been imaged and we only observe quenching once a sufficient power P is applied (see Fig. 2 and Fig. 3), we can definitely claim that the invasiveness of the measurement in our case is insufficient to break superconductivity.

In response to Reviewer #2 (point 1), we note that we did not observe any change in the vortex shape or pattern when measuring with different fast axes (row-wise or column-wise scans), from which we conclude that there is no vortex dragging to be observed at the spatial resolution we achieve.

In response, we have added the following paragraph to the “Magnetometry measurements” section in the methods. We also added new Ref. 48: Johnson, PRL, 1991.

[...] *To read out the spin state of the NV optically, the beam of an in-house-built green laser (520 nm [19]) is focused on the NV center and the fluorescence is collected. Consequently, due to the size of the focused beam and the proximity of the NV to the sample, some laser light reaches the sample. This can be observed from the heating of the sample when operating at mK temperatures. However, the heating power of the microwave pulses (~300 µW) is significantly larger than the laser power when pulsed (~50 µW) [19].*
Generally, the laser is only on for a short duration of time (~1 µs) as the ODMR sequence is performed in a pulsed manner. The laser initialization/readout pulse and the microwave π-pulse are separated by a dark interval of ~1 µs in our pulse sequence, while quasiparticle relaxation times are on the order of <1 ns [48].
This means that the superconductor has sufficient time to recover from the previous illumination and is effectively not affected besides the global heating, which is well understood [19].
Additionally, because Meissner screening of the full device has been imaged and we only observe quenching once a sufficient power P is applied (see Fig. 2 and Fig. 3), the invasiveness of the measurement in our case is insufficient to break superconductivity.

3. What is the pattern and time scale of the change in the turn-on voltage? How often does this voltage change? And how long does it take to obtain a magnetic field map? In figure 3b, if you extrapolate both datasets to $r_0 = 0$, then you could define a reasonable turn-on voltage in this case

(x-intercept) at the two different temperatures. Since it is known that it is time-consuming to obtain a magnetic field scan, I am assuming the time scale of the whole dataset should be on the order of days if not weeks. How could the extrapolated turn-on voltage be so stable over such a long time, which seems to contradict what the authors report in figure 1c for the fluctuating turn-on voltage measured with electronic transport.

Authors: The pattern and time scale of the change in the turn-on voltage can be inferred from supplementary Figure S2, which gives a time trace of the gate voltage (given in units of gate power $P=V*I$, at constant current I = 100 nA). The time traces show that fluctuations occur on the time scale of minutes to hours and can be abrupt. In response, we now also plot the time trace in units of the gate voltage in Figure S2.

Regarding the apparent contradiction between the stability in Figure 3b and the fluctuations in Figure 1c: The selection of IV curves in Figure 1c deliberately shows a large span of different onset voltages on device D2b, measured over multiple months. Figure 1c is meant to represent the full range of onset voltages we observed over the measurement campaign.

In contrast, Figure 3b plots the time-averaged gate voltage for each data point. Since magnetometry scans take several hours to complete, we continuously record the gate voltage and use the mean value for the x-axis in Figure 3b. We realize that this was not clear from the Figures (3b and S4c), which has now been corrected in the figures and in the respective captions. We have further added error bars to the voltages indicated in Figure 3b, corresponding to the standard deviation of the recorded gate voltage during each scan.

4. Following the previous point, the authors' interpretation to the data in figure 3b is confusing. The authors claim that they do not see a correlation between gate voltage and their extracted quenching radius, thus ruling out the possibility of direct electric field effect. However, it is pretty probable from figure 3b that the relation between gate voltage and r0 could be linear. In addition, the fact that you could obtain a well-defined turn-on voltage that is stable over all experiments based on the x-intercept for a linear fit to each dataset probably suggests a minimum electrical field for GCS, thus contradicting the authors' claim that they rule out electric field as a source of the quench. Moreover, figure 3b shows that the turn-on voltage is lower at lower temperature, suggesting that temperature also plays some role here. The model that the authors present provides a fit to the data in figure 3a, yet the conclusion that the authors draw from this model seems to disagree with what figure 3b tells. The authors should address this discrepancy in detail to justify their claim that GCS is independent of electric field and temperature.

Authors: We agree that Fig. 3b alone is not fully conclusive in supporting the claim that "there is only a weak correlation between gate voltage and the extent of the Meissner state suppression". The seemingly linear relationship, and even the dependence on temperature, are partially coincidental. This can be seen when taking the second dataset (Fig. S4c) into account, where r_0 and V_G are clearly uncorrelated, and there is no obvious temperature effect. We only show the data of Fig. 3b in the main text to stay consistent with Fig. 3a. However, the dataset of Fig. S4c would be more suited to answer the Reviewer's concern. Furthermore, we have added a supplementary figure that shows consecutive scans at the exact same gate voltage, but completely different suppression states (Figure S8).

In the revised manuscript, we now point out in the main text that Fig. 3b is not fully conclusive and that dataset of Fig. S4c is needed to fully support independence of r_0 from V_G and T. We also reference the newly added supplementary figure S8.

The manuscript has been revised as follows (page 3, right column):

"Finally, a direct electric field effect, without a leakage current, is also excluded because the same gate voltage can result in different quenching radii, shown in Fig.S4c. This occurs when the I_{leak} vs. V_{gate} relationship changes. Moreover, Fig. S8 shows two consecutive measurements that exhibit completely different suppression states at the exact same gate voltage."

5. The authors show data in figure 4 for different gate configurations. The authors should consider carry out the same quantitative analysis for at least one more different gate geometry, for example, G2/3. If the source of the hot carriers is correlated with the point with the highest current density as the authors claim, then that should show up as a much lower \alpha in their model for G2/3 since the electrode geometry is more spread out. The author should provide this additional dataset to support their claim, and explain how they expect the parameter \alpha to scale with respect to other factors such as electric field intensity or current density. That being said, the author should address point 2 and 3 first to justify the main claim of this work.

Authors: We thank the reviewer for this insightful comment. We agree that a quantitative analysis of the suppressed region around spread-out gates G2/G3 and point like gate geometries G1/G4/G5 is a good way of testing the hypothesis of spatial correlation between the current density and NEP hot spots.

On the extraction of α for G2/G3: The reviewer suggests extracting the parameter α for the extended gate geometries (G2/G3) to see if it is lower. However, we respectfully note that our current model is not suited to extract α for these geometries. The analytical model derived in the manuscript (Equation 3) is strictly a solution to the diffusion equation for a point-like source. Since the gates G2/G3 have a length of ~2.6 µm (which is significantly larger than the extracted NEP diffusion length r_d = 0.6 µm), the gates act as a line source. Qualitatively, this confirms the hot-spot model – because of the extended length of the gates, the NEP generation is spread over an extended length, leading to a lower NEP density and therefore smaller region of suppressed superconductivity.

To further address the concern raised by the reviewer (i.e., that the geometry should influence the quenching efficiency), we have directly compared the quenching extent of the different gate geometries at fixed gate power. This comparison is shown in supplementary Figure S12 for gates G2 and G4. For the point like gate G4, the suppression penetrates deep into the island, yielding a maximum quenching width of w_{G4} = 1.23 µm. In contrast, the suppression is confined to a shallow strip along gate G2 at the same gate power, yielding a maximum quenching width of w_{G2} = 0.28µm. The quenching distance for the point like source is thus roughly 4x times larger than that of the extended source. This agrees well with the hypothesis that the spread-out leakage current at gate G2 results in a much lower local current density, creating a lower local density of NEPs and a significantly reduced suppression range compared to the concentrated injection at gate G4.

6. Could the authors please explain how they identify that there are four superconducting vortices in figure 2c? I do not think I know how to read from that map that there are vortices and their

positions. Also, would the vortices reappear at the same positions if the sample is thermal-cycled (heated above critical temperature and cooled down again)?

Authors: We now indicate the vortices in Fig. 2c. The vortices are visually identified and agree with the calculated amount of flux, see Ref. 45 [P. Scheidegger, PhD Thesis No. 30465, ETH Zurich (2024)], Section 6.3. In addition to this, we have added Figures S9 and S10 to the supplementary information; these figures showcase respectively vortex behavior after thermal cycling and the reproducibility of vortices after applying large gate powers within the same cooldown.

****
Reviewer #2 (Remarks to the Author):

The manuscript is focused on imaging the stray magnet field properties of thin film superconducting niobium under the influence of a local gate electrode using nitrogen-vacancy center magnetometry at sub-kelvin temperatures.
The authors argue that they are able to determine the origin of "gate controlled superconductivity" (GCS) via measurements of the Meissner effect in regions of the superconducting film that are near a local gate. They are argue that the GCS effect results from current leakage between the gate and the superconductor, which injects hot quasiparticles that then quench superconductivity in a circular region whose radius reflects a diffusion length.
These are an impressive series of experiments using scanning NV magnetometry at sub-kelvin temperatures, which provide direct imaging of superconducting characteristics and strong support for the hot quasiparticle injection mechanism of GCS. These measurements also provide quantitative insight into GCS that is largely inaccessible via conventional transport measurements. As such, I believe that the results could be broadly impactful to mesoscopic superconductivity and superconducting technologies. Before recommending for publication, I believe the authors should address a few outstanding issues identified below.

Authors: We thank the Reviewer for taking their time to assess our manuscript.

1. The effect of the laser, required for optical readout of the nitrogen-vacancy center, is not discussed. This is an important limitation of NV center magnetometry that needs to be explicitly addressed particularly in this study.
The underlying mechanism for hot quasiparticle injection and complete quenching of superconductivity via gate leakage appears to depend on power dissipated through the gap between superconducting islands (~ 1 microwatt or less in a ~100nm squared area associated with the gap between superconducting islands). To my knowledge NV center readout requires up to two orders of magnitude higher laser power (probably around 100 microwatts), which in this scanning experiment is focused tightly onto the measurement location via the scanning probe pillar presumably something like 100 nm in diameter. The power density associated with the laser therefore appears to far exceed that associated with the gate leakage. I am aware that the mechanism of power absorption is different in the case of gate leakage versus optical illumination. However, by this argument of power density, I would think that the laser would have a huge effect on the stray field data as it moves around the superconducting film during a tip raster scan – locally creating its own hot spot of non-equilibrium quasiparticles underneath the measurement region.
This effect could be relevant to the observed suppression of Meissner screening in the bulk film in Fig. 4. The laser may also drag around vortices in cases where they are present like in Fig. 2A. I am also wondering to what extent the laser extrinsically "expands" the radius of quenched

superconductivity around the gate by adding an additional source of non-equilibrium quasiparticles, which would affect the quantitative analysis around Fig. 3.
The authors should state the laser power used, the width of the diamond pillar, whether it is a continuous wave or pulsed measurement, and include some arguments about the extent they believe the laser is or is not affecting the results, as well as any steps they took to mitigate or understand the possible invasive effect of the laser. It could be a supplementary section that is referenced in the main text.

Authors: Please see our response to Reviewer #1, point 2, who raised similar questions.

2. In reading the main text and supplement, it is very difficult to find out how exactly the values of r0 and their error bars are obtained in Fig. 3. This appears to be a critical piece of analysis in the present manuscript, but the text describing these methods in both the main text and supplement are confusing.

Authors: In Fig. 3, the r_0 is obtained using the 'second approach' (which finds the extrema) for D3. This also holds for Fig. S4 for D2a and D2b. The reason that both methods are mentioned is that some parameters, like the stand-off height and the London penetration depth, are only extracted using the 'first approach'. The results for r_0 extracted with the 'first approach' are presented in Fig. S3.

a. There are two separate analysis methods brought up in the main text when only one is used to obtain the values of r0 presented in Fig. 3. The main text is also unclear about exactly which method is used to obtain the presented r0 values. It seems that "approach 2: finding extrema" is the one used for Fig. 3, but this is not at all clear from reading the main text.

Authors: We have rephrased the main text: "*The results of the analysis following the second approach are presented in Fig. 3a for device D3*."

b. The description of the relevant "approach 2" in the main text is too vague "The second approach involves locating the field step in a Bx map and determining the shift relative to a reference map measured with VG = 0". What is the shift they are referring to? What is a field step?

Authors: The field step refers to the extrema in the B_x in case of D2b/D2b (Figure S4b/c) or extrema in $\partial B_x/\partial x$ in case of D3 (Figure 3a/b). The extrema coincide with the edge of the device, as described in Supplementary Section 1.2. By tracking the relative shift of these extrema due to the GCS effect compared to the case that the source meter unit is off (V_G = 0), we find the area that is suppressed.

We made the description of the second approach more specific in the main text:

"The second approach involves the row-wise location of extrema in maps of B_x (D2a and D2b) or $\partial B_x/\partial x$ (D3, see Fig. S4d), which align with the edge of the superconducting domain [ref Supplementary Information]. We quantify the quenching radius r_0 by extracting the shift of these row-wise extrema relative to their position in a reference map at V_{gate}=0 [ref. Supplementary Information."

Can the authors state, in the main text, the exact analysis procedure that is used on device D3, list the fit parameters involved in this analysis, and state explicitly which parameters are are kept fixed as the gate power is varied? It could be useful to incorporate some version of Fig. S4D into Fig. 3. It is only after seeing Fig. S4D that I could understand what the actual free parameters are in this procedure and how r0 is reflected in the raw data.

Authors: We thank the reviewer for their comment about clarifying the analysis procedure that went into the creation of Figures 3a and b. However, we think that the detailed description of how the quenching radius r_0 is obtained from the scans should remain in the supplementary section, as the full picture is very technical and does not add to the understanding of the GCS effect. We hope that the changes in the description of the second approach to extracting r_0 as well as the direct reference to Figure S4d in the main text will give readers an improved understanding of the applied method, while not distracting them from the main findings of the quantitative analysis.

c. The error bars in r0 in "approach 2" are obtained via Monte Carlo simulation according to the supplement, but no further information is provided. This needs to be explained in more rigorous detail.

Authors: We have expanded Supplementary Section 1.1.2 to provide a more detailed explanation of the Monte Carlo uncertainty propagation, where we explain how the uncertainties in the offset d and the extracted quenching area A_q were propagated by generating a distribution of quenching radii: *"Both of these errors were propagated using a Monte Carlo simulation to determine the error bars of r_0 in Fig. 3a. In particular, we generated normal distributions of $X_d \sim N(d, \sigma_d)$ and $X_{Aq} \sim N(A_q, \sigma_{Aq})$, from which we drew N=1000 random samples $\{d^i, A^i q\}_i$. For each sample pair, we calculate the corresponding quenching radius r_{0i} by inverting equation S1. The error bars of r_0 correspond to the standard deviation of the resulting sets $\{r^i{}_0\}_i$."*

3. "Curiously, near zero gate power, we observe a cluster of four superconducting vortices within the Nb island"
Can the authors describe in more detail, or label on the figure what features they are associating with four vortices? This is done in the supplement, and it would be useful to do the same in the main text.

Authors: We now indicate the vortices in Fig. 2c [see also Reviewer #1, point 6].

4. Overall, I find it hard to discern what is actually the independent variable in many of the data shown in the manuscript.
a. The methods state that transport data in Fig. 1 are taken in "constant voltage" mode, whereas the caption of Fig. 1 states that it is taken in "constant current" mode.

Authors: Unless stated differently in the text related to the figure/caption of the figure, the I/V curves have been measured in the constant voltage mode, while the magnetometry experiments have been measured with the constant current mode (which was effectively turned into a constant power mode with an outside loop for most of the magnetometry scans of device D3). These points have now been clarified in the methods section of the manuscript.

We have also rephrased the caption in Figure 1:

"In constant voltage mode, a voltage V_G is applied between island and gate, while measuring leakage current I_L. Conversely, in constant current mode, a leakage current I_L is applied, while the voltage V_G can be measured. Scale bar, 1 µm. c, I_L/V_G curves measured on device D2b in constant-voltage mode."

b. The methods also state that magnetometry measurements on D3 are performed with the SMU in constant current mode, where the current is dynamically adjusted to keep the power fixed. Other magnetometry data is taken in standard constant current mode.
Throughout the manuscript, however, the scans are labeled or referred to by gate voltage "VG" (e.g. in Fig. 3b, and the surrounding text, as well as in related supplemental figures). Based on the methods, however, VG is never the independent variable in the magnetometry scans. It is either I_L or power, depending on whether the authors used the PID loop to keep power fixed or not. The figures and text should accurately reflect the independent variable which is controlled in the experiment.

Authors: Indeed, the gate voltage is never the independent variable during the magnetometry scans. We have adapted the figure labels and surrounding text to clarify this point. We also indicate that the reported gate voltage reflects the mean voltage recorded during the magnetometry scans. We have further added error bars to the plotted voltages in Figure 3b, indicating the standard deviation of the recorded voltage during each scan (they are only visible for a few datapoints, due to the low standard deviation of the gate voltage during these scans).

5. How are the points labeled by VG in Fig. 3b? It appears to be the same data as in Fig. 3a, but replotted with a different x-axis. If power is being held fixed during each scan, VG could be changing by up to several volts during this procedure (I am inferring this from the transport data in Fig. 1). Therefore each r0 measurement is not associated with a single value of VG. Does the x-axis of Fig. 3b represent some "average" or "nominal" gate voltage over the course of a scan? If so, there should be an indication on the plot of the spread in VG for each data point.

Authors: In Figure 3b, the gate voltage is averaged over the entire scan. We realize that this was not stated in the figure caption, which is corrected now [See also Reviewer #1, point 3]. We have also added error bars to Figure 3b to indicate the spread of the voltage during each magnetometry scan.

6. "Finally, a direct electric field effect is also excluded because the quenching is not correlated with the gate voltage (Fig. 3b)"

The quenching does appear to be correlated with VG. Do the authors mean that they are not linearly correlated, and this excludes direct electric field effects? I would contest this given point 4 above – each extracted r0 data point does not have a single VG associated with it, so how can the authors conclude how r0 scales with VG?

Authors: Please see our response to Reviewer #1, point 4, who raised the same question.

7. The thermal diffusivity constant quoted for glass appears to be a room temperature value if I am not mistaken. This is probably not quantitatively relevant to the present experiment.

Authors: Indeed, it was accidentally the room temperature value. This has now been adjusted in the main text:

"For substrate diffusion, we calculate D $\sim 5 \cdot 10^{-4}$ m^2/s (from the values for amorphous silica [29, 30]), yielding a similar value as for quasiparticle diffusion."

We used specific heat *c* and thermal conductivity *K* from [29] and density ρ from [30] and calculated the diffusion constant *D* using the equation:

$D = K/(\rho * c)$

(from NIST: https://www.osti.gov/servlets/purl/761710-u8kdDW/webviewable/)
Specific heat *The*(4K) = 0.1 J/(kg K) {read out from graph page 104},
Thermal conductivity *K*(*T*) = 0.1 W/(m K) {table interpolated value between 3 K and 5 K}

The density is assumed to be constant over temperature
(from: https://ieeexplore.ieee.org/stamp/stamp.jsp?tp=&arnumber=1479653)
Density ρ = 2200 kg/m^3 {table on page 1249}

8. "For quasiparticle diffusion, D ~ 5·10−4 m2/s"
It should be stated explicitly that this value corresponds to quasiparticles in niobium thin films as inferred from transport measurements.

Authors: We rephrased the main text:

"For quasiparticle diffusion, using D $\sim 5 \cdot 10^{-4}$ m^2/s obtained from transport measurements in thin film niobium [13] yields a lifetime of $\tau = r_d^2/D \sim 0.7$ ns. "

9. Throughout the manuscript the authors refer to a London penetration depth. The length constant obtained via the stray field should be a Pearl length relevant for thin film superconductors. Are they converting the Pearl length to London penetration depth? If so, they should state how this is done.

Authors: We are directly fitting for the penetration depth, which is used in the SuperScreen package to calculate the effective penetration depth, as noted in the Methods section ($\lambda_{\text{eff}} = \lambda^2/d$). This would be the Pearl length/2.

****
Reviewer #3 (Remarks to the Author):

The paper by Scheidegger et al. reports imaging of Nb islands subjected to a large leakage current via scanning NV magnetometer. Various observations are reported on how the leakage current affects the superconducting state.
In my opinion, the paper shows limited amount of novelties for a Nature journal. The main novelty is that a leakage current suppressed Meissner screening. However, this is not extensively characterized, and it is based only on one thin film (Nb, 20 nm). Instead of simply showcasing the observation, the authors should understand it better, and in different films. If further understanding would be gained about this point I would reconsider my opinion.
Other key conclusions are all well-known facts. It is known that power has most effect (if voltage is sufficiently large). It is known that the impact on superconductivity rapidly decreases with distance. It is also known that the effect takes places via generation of a hot spot and spreading of phonons. I do not see significant breakthroughs with respect to these known facts.

Authors: We thank the Reviewer for taking their time to assess our manuscript.

We respectfully disagree with the Reviewer's opinion of *limited novelty* of our work. While we agree that recent literature has shown that leakage currents and subsequent phonon/quasiparticle injection are in many instances the primary drivers of the GCS in superconducting structures similar to the ones we studied, our experiments demonstrate, for the first time, that the effect also manifests in the suppression of the Meissner screening. By imaging the Meissner effect, we provide the first direct evidence of how the superconducting state (and not just the switching current) is spatially quenched.

Further, we do not only showcase the observation but also extract quantitative numbers for e.g. the diffusion length and spatial symmetry of the effect. We are not aware of prior literature on this. Furthermore, it is not clear yet to which extent the GCS effect is related to power, neither has the mechanism via phonons been settled.

Finally, while the Reviewer is correct that we only looked at one material combination (Nb on SiO_2), we did observe the GCS on three different devices, stemming from different fabrication rounds, over multiple thermal cycles with different (gate) geometries. Thus, we think that our observations and conclusions are well founded.

Questions and comments

What happens to the vortexes after the voltage is set back to zero? Do they appear again, or they are gone forever?

Authors: The vortices reappear at the same place, see Fig. S10 that has been added to the supplementary. Next to this, the effects of thermal cycling are shown in the new supplementary Fig. S9.

The paper starts with a fake news: "It was recently discovered that supercurrents flowing through thin superconducting nanowires can be quenched by a gate voltage." In fact, a very strong and large body of work indicates that the gate voltage has no effect on superconductivity in metals.

Authors: The phrasing is chosen to be in line with the initial report of the GCS effect by De Simoni et al. in 2018 (Ref. 1), and it does not make a statement about the absence or presence of a leakage current. Our statement also does not contradict the Reviewer's second statement that the gate voltage alone may have no effect. In fact, it is in line with the findings of our study.

In response, we have clarified the phrasing in the main text:

"It was recently discovered that supercurrents flowing through thin superconducting nanowires can be quenched by *applying a voltage to a nearby gate electrode*."

This sentence summarizes the experimental observation, with no prejudice on the underlying microscopic mechanism.

The acronym GCS is wrong, and I suggest to stop using it. Tt is not gate controlled, and what is controlled is the switching current, not the supercurrent.

Authors: We refer to the phenomenon using the acronym GCS that has been established in the literature, because we study the complementary aspect of superconductivity, namely the diamagnetic response of devices subject to a gate voltage.

The GCS phenomenon has been documented by a large body of literature, reviewed in Ref. 6 [Ruf et al, Appl. Phys. Rev. 11, 041314 (2024)], and its existence is a robust fact. What is less established is the exact microscopic mechanism – for example, there is strong evidence that it is not due to an electric-field effect related to the application of a gate voltage (what is referred to in the literature as "direct field effect"), but instead a secondary mechanism triggered by the application of the gate voltage. In our devices, the suppression of the Meissner effect is clearly not a direct field effect, but a leakage-current related effect. Experimentally, the GCS effect is caused by applying a gate voltage (whatever the microscopic mechanism), which is adequately reflected by the acronym.

Second line of the paper: "owing to the possible new physics ". New physics does not mean anything.

Authors: We note the Reviewer's reservation against the cited wording, which is nevertheless widely used in literature to express undiscovered or unexplained physical phenomena or mechanisms. We are aware of the fact that meanwhile it has been understood that most GCS observations can be explained by a leakage current related mechanism. Still, our statement is correct that the possibility of revealing "possible new physics" was a driving force for the field.

In response, we rephrased the sentence as follows:

"The recent discovery of a gate-controlled supercurrent (GCS) in superconducting nanoconstrictions [1] has sparked significant scientific interest, owing to the *unexpected observations* and to potential applications in transistors for low power dissipation electronics [2–5]."

Another wrong fact appears right afterwards: "A defining feature of the GCS is the complete switching of a typically small superconducting volume into its normal state under the application of a sufficiently large gate voltage." Of course the gate voltage is not doing anything here. Second, I am not aware of evidence that reports the complete switching of superconductivity. In fact most reports show that the switching current goes to zero, not that superconductivity is quenched. I am concerned by the fact that the authors spread this unsupported information as "defining fact".

Authors: We agree that our original wording was imprecise. As detailed already in our response above, by just measuring the charge transport, one cannot assess the thermodynamic state. This is exactly the reason why studying the Meissner effect is important. Our results presented in this manuscript indeed show that the GCS is more than just suppressing a transport current.
In response, we rephrased the sentence as follows:
"A defining feature of the GCS is the complete switching of a typically small superconducting *region from its zero-resistance state to the normal-resistance* state under the application of a sufficiently large gate voltage."

Finally "Although significant work has been put into developing a microscopic understanding of the GCS effect, its precise origin is still under debate." This is completely false, and it is likely written to make the paper more appealing. Everybody knows the origin of such effect since 2021-2022. There is no doubt in the community that there is no electric field involved.

Authors: We disagree. There are conflicting experimental observations, and while some mechanisms are more probable than others, they are still under debate. Even when excluding the electric field as a possible mechanism, there remain several and in parts conflicting models about how a leakage current can give rise to the GCS observations. We refer the Reviewer to [Appl. Phys. Rev. 11, 041314 (2024); Section III]. We are of the opinion that our statement adequately reflects the state of the art.

Reviewer #4 (Remarks to the Author):

>> I co-reviewed this manuscript with one of the reviewers who provided the listed reports. This is part of the Nature Communications initiative to facilitate training in peer review and to provide appropriate recognition for Early Career Researchers who co-review manuscripts.

Authors: We thank the Reviewer for taking their time to assess our manuscript

****
Summary of changes made to the manuscript:

Author list:
We reordered the author list. P. Scheidegger (previously first author) is now equally-contributing third author.

Abstract
It was recently discovered that supercurrents flowing through thin superconducting nanowires can be quenched by *applying* a gate voltage *to a nearby gate electrode*.

Introduction
The recent discovery of a gate-controlled supercurrent (GCS) in superconducting nanoconstrictions [1] has sparked significant scientific interest, owing to the *unexpected observations* and to potential applications in transistors for low power dissipation electronics. A defining feature of the GCS is the complete switching of a typically small superconducting *region from its zero-resistance state to the normal-resistance state* under the application of a sufficiently large gate voltage.

Results—Superconducting island devices
Figure 1bc caption:
b [...] *In constant voltage mode, a* voltage V_G is applied between island and gate, *while measuring leakage current* I_L. *Conversely, in constant current mode, a leakage current* I_L *is applied, while the voltage* V_G *is measured*. Scale bar, 1um.
c I_L/V_G curves measured on device D2b *in constant voltage mode*.

Results— Quantitative analysis of quenching
[...] However, because this method does not account for vortices, it cannot be applied to the entire dataset. *The second approach involves the row-wise location of extrema in maps of* B_x *(D2a and D2b) or* $\partial B_x/\partial x$ *(D3, see Fig. S4***d***), which align with the edge of the superconducting domain [25]. We quantify the quenching radius* r_0 *by extracting the shift of these row-wise extrema relative to their position in a reference map at* $V_{gate}=0$ *[25].* The results of *the analysis following the second approach* are presented in Fig.3a for device D3. The dataset combines measurements at two temperatures

(0.4K, 5.6K) and at positive and negative gate voltages (*i.e. both gate polarities*) collected during a single cool-down.

Figure 3b caption:

b, Data of **a** plotted against *the average* gate voltage $|\overline{V}_G|$ showing only weak correlation thereby ruling out a direct field effect (*see also Fig. S4**c***).

[...] *Finally, a direct electric field effect, without a leakage current, is also excluded because the same gate voltage can result in different quenching radii, shown in Fig. S4**c**. This occurs when the I_{leak}/V_{gate} relationship changes. Moreover, Fig. S8 shows two consecutive measurements that exhibit completely different suppression states at the exact same gate voltage.*

Hot-spot model

In the following, we argue that the quenching of superconductivity is caused by non-equilibrium particles (NEPs)—namely, hot electrons or phonons [6, 13, 16, 17, 20]*, created by the leakage current.* The suggested microscopic mechanism is sketched in Fig. 3c: the NEPs, generated at the location of highest leakage current density (ie., below the gate apex), form a local hot spot. This hot spot is similar to that observed in superconducting single photon detectors, however, it is not caused by heating [26], *and the number of excited NEPs can exceed the number of charge carriers of the leakage current by far [14, 26]. Because of the relatively large distance between gate and Nb island, we argue that the NEPs reaching the island are mainly phonons*. When impinging on the superconductor, the NEPs excite quasiparticles by breaking up Cooper pairs, leading to a suppression of superconductivity. While diffusing, both types of particles—*phonons in the substrate and quasiparticles in the superconductor*—also undergo thermal relaxation, eventually limiting the radius of the suppression.

For quasiparticle diffusion, *using* $D \sim 5 \cdot\cdot 10^{-4}$ m^2/s *obtained from transport measurements in thin film niobium* [29, 30] *yields* a lifetime of $\tau = r_d^2/D \sim 0.7$ ns. This is roughly ten times longer than the time for hot-spot formation reported in [13] and within the order of magnitude of superconducting single photon detector recovery [28].

For substrate diffusion, we calculate $D \sim 5 \cdot\cdot 10^{-4}$ m^2/s *(from the values for amorphous silica [29, 30]), yielding a similar value as for quasiparticle diffusion. The similarity between these two transport scales prevents a purely quantitative assignment of the diffusion to one specific medium.*

Methods—Magnetometry measurements

[...] *To read out the spin state of the NV optically, an in-house-built green laser (520 nm [19]) is shone on the NV and the fluorescence is collected. Consequently, due to the size of the focused beam and the proximity of the NV to the sample, some laser light reaches the sample. This can be observed from the heating of the sample when operating at mK temperatures. However, the heating power of the microwave pulses (~300 µW) is significantly larger than the laser power when pulsed (~50 µW) [19]. Generally, the laser is only on for a short duration of time (~1 µs) as the ODMR sequence is performed in a pulsed manner. The laser initialization/readout pulse and the microwave \pi-pulse are separated by a dark interval of ~1 µs in our pulse sequence, while quasiparticle relaxation times are on the order of <1 ns. This means that the superconductor has sufficient time to recover from the previous illumination and is effectively not affected besides the global heating, which is well understood [19].*

Additionally, as Meissner screening of the full device has been imaged and we only observe quenching once a sufficient power P is applied (see Fig. 2 and Fig. 3), we can definitely claim that the invasiveness of the measurement in our case is insufficient to break superconductivity.

****

Reviewer #1 (Remarks to the Author):

The authors have made several changes to the manuscript that address most of our questions; in particular their argument about phonon diffusion leading to Cooper pair breaking is reasonable. I would suggest, however, that their assertion that they cannot distinguish whether the diffusion length is related to phonon propagation or quasiparticle diffusion merits some more quantitative attention. Since their model assumes phonon propagation in the substrate, the authors should be able to give an estimate on the characteristic length of phonon propagation/dissipation given that the substrate is SiO2/Si stack, whose properties are well-known. We believe they could also easily estimate the diffusion of quasiparticles in Nb, whose lifetime is probably a well-known quantity as the authors cited in their response to the next question. Thus, we would encourage the authors to provide an estimate of the relevant length scales and compare that to their measurement.

Authors: We thank the Reviewer for raising this point, which has made us rephrase the interpretation of the hot-spot model in terms of phonon/quasiparticle diffusion.

To start, the diffusion occurs in both the substrate and the superconductor. For quasiparticle diffusion in the superconductor, we can use D ~ 5e-4 m^2/s obtained from transport measurements in thin-film niobium [Jalabert 2023] and the quasiparticle scattering time τ (ranges between 0.1-1 ns in the literature, we take an average value τ ~ 0.5 ns from [Leo 2011]) to estimate $l = (D*\tau)^{1/2}$ ~0.5 um. This estimate is remarkably close to our experimental measurement. This fact does not prove the diffusion mechanism, but it strongly suggests (corroborated by the excellent fit of Fig. 3a) that quasiparticle diffusion is the dominant process in our measurements.

Diffusion for phonons is much harder to estimate, because (i) the silicon oxide layer (300nm thick) is amorphous, (ii) the underlying crystalline silicon cannot be neglected, and (iii) phonons responsible for breaking Cooper pairs have very high energies such that literature values for the thermal conductivity do not apply. Nevertheless, computing the diffusion coefficient from literature values ($D = \kappa/(c*\rho)$, where κ is thermal conductivity, c specific heat and ρ density) we find D ~ 5e-4 m^2/s, coincidentally the same value as for the quasiparticles. However, we think this D grossly overestimates phonon diffusion, because scattering of high frequency phonons is much enhanced, and because of the silicon substrate. Moreover, κ (and thus D) strongly depend on temperature, while our measurements show no temperature dependence. Together, this points towards a minor contribution of phonon diffusion to the quenching radius r_0. However, we also note that *some* phonon diffusion does occur, as this is needed to explain Fig. 4d/e.

In response, we have rephrased the paragraphs interpreting the hot-spot model to reflect the above discussion.

A more serious remaining concern: regarding our questions about the claimed lack of correlation between gate voltage and suppression of superconductivity, we still find the authors' explanations confusing. If the proposed mechanism arises from leakage current -> phonons -> quasiparticles, then shouldn't the same gate voltage give the same leakage current, and thus give reproducible suppression (comparing S4c to S8)? More to the point, there should just be a clear quantitative relationship, as well as an empirical I-V curve, that relates leakage current to applied voltage, and predicts the correlation between r and Vg.

Authors: No, the same voltage does not always yield the same leakage current due to the VSILC (variable stress-induced leakage currents) effect as mentioned in the paper (Section "leakage currents" in Methods). This is in line with what we observe: the varying I-V curves, as shown in Figure 1c. This can also be observed in Figure S2b, where a 100 nA constant current is applied and the gate voltage is changing. We acknowledge that this might not have been clear enough around Figure S2b and therefore, we have now added "while applying I_L = 100 nA in constant current mode" in the caption.

Reviewer 2 question 2 made a fair point that the interpretation of the errorbar in figure 3 is somewhat confusing and it lacks detailed explanation on how they are obtained. The authors should state clearly in the caption what the error bars stand for, or point to the relevant section in the SI where detail is provided.

Authors: We have updated the caption of Figure 3 to state that the error bars represent the standard deviation of the quenching radius r_0, obtained by propagating experimental uncertainties through a Monte Carlo simulation. We also provide a reference to the supplementary section, where the experimental uncertainties and the simulation parameters are discussed in detail.

****
Reviewer #3 (Remarks to the Author):

The paper improved significantly in the wordings, and I thank the authors for reflecting on my comments. When the authors claim "suppression of superconductivity", it is unclear if they mean that screening is absent, the sample turned resistive, or it turned normal. I still believe that GCS is the wrong wording, but I will not complain further on this point.

I find the novelty of this work still limited, as most of their findings were already well known. The observation that injection of high energy electrons suppresses screening is new and interesting. Overall, I favor publication.

Authors: We thank the Reviewer once more for taking their time and for their positive assessment.

****
Reviewer #4 (Remarks to the Author):

>> I co-reviewed this manuscript with one of the reviewers who provided the listed reports. This is part of the Nature Communications initiative to facilitate training in peer review and to provide appropriate recognition for Early Career Researchers who co-review manuscripts.

Authors: We thank the Reviewer once more for taking their time to assess our manuscript.

****
Summary of changes (a PDF with markup "manuscript_diff.pdf" is included in the resubmission)

Manuscript:
- Fig. 3 caption: We now give a brief explanation of how the error bars were obtained, and refer to the supplementary section.

- We modified the last two paragraphs of the section on the “hot spot model” to better relate our measurement to the expected diffusion lengths in Nb resp. SiO/Si.
- We updated the Author Contributions and Acknowledgement sections reflecting the extra work performed during revision.
- We made some cosmetic changes to the manuscript text (see PDF markup)

Supplementary:
- Fig. S2c: we clarified that this measurement was taken in constant current mode.
